# Reactive Pulsed Laser Deposition of Clustered-Type MoS*_x_* (*x* ~ 2, 3, and 4) Films and Their Solid Lubricant Properties at Low Temperature

**DOI:** 10.3390/nano10040653

**Published:** 2020-04-01

**Authors:** V. Fominski, M. Demin, V. Nevolin, D. Fominski, R. Romanov, M. Gritskevich, N. Smirnov

**Affiliations:** 1 National Research Nuclear University MEPhI (Moscow Engineering Physics Institute), Kashirskoe sh., 31, 115409 Moscow, Russia; nevolin@lebedev.ru (V.N.); dmitryfominski@gmail.com (D.F.); limpo2003@mail.ru (R.R.); mgritskevich@yandex.ru (M.G.); 2 Immanuel Kant Baltic Federal University, A. Nevskogo St 14, 236016 Kaliningrad, Russia; sterlad@mail.ru; 3 Mechanical Engineering Research Institute of the Russian Academy of Sciences, Bardina St 4, 119334 Moscow, Russia; smir1947@yandex.ru

**Keywords:** reactive pulsed laser deposition, solid lubricants, nanoclusters, molybdenum sulfides, coefficient of friction, wear, low temperature

## Abstract

We studied the tribological properties of amorphous molybdenum sulfide (MoS*_x_*) thin-film coatings during sliding friction in an oxidizing environment at a low temperature (−100 °C). To obtain films with different sulfur contents (*x* ~ 2, 3, and 4), we used reactive pulsed laser deposition, where laser ablation of the Mo target was performed in H_2_S at various pressures. The lowest coefficient of friction (0.08) was observed during tribo-testing of the MoS_3_ coating. This coating had good ductility and low wear; the wear of a steel counterbody was minimal. The MoS_2_ coating had the best wear resistance, due to the tribo-film adhering well to the coating in the wear track. Tribo-modification of the MoS_2_ coating, however, caused a higher coefficient of friction (0.16) and the most intensive wear of the counterbody. The MoS_4_ coating had inferior tribological properties. This study explored the mechanisms of possible tribo-chemical changes and structural rearrangements in MoS*_x_* coatings upon contact with a counterbody when exposed to oxygen and water. The properties of the tribo-film and the efficiency of its transfer onto the coating and/or the counterbody largely depended on local atomic packing of the nanoclusters that formed the structure of the amorphous MoS*_x_* films.

## 1. Introduction

Friction-related reductions in energy consumption in friction joints and the prevention of component wear in these joints under adverse conditions (e.g., low temperatures, aggressive/oxidizing atmosphere) are important research problems in space engineering, cryogenics, micromechanics, and other fields [1,2,3,4]. These problems are solved by using organic/liquid lubricants or by applying solid lubricant coatings if liquid lubricants cannot be used. The most-studied tribological properties (under adverse low-temperature conditions) are molybdenum disulfide (MoS_2_) solid lubricant coatings [5,6,7]. A decrease in temperature during sliding friction in a vacuum did not have a noticeable effect on antifriction properties; moreover, it can reduce the volume of material removed due to wear [8]. The tribological properties of MoS_2_-based coatings can, however, significantly worsen under the influence of aggressive components (oxygen) fed into the vacuum chamber holding the sample [9,10].

The tribological properties of MoS_2_-based solid lubricant coatings and other transition metal dichalcogenides have been extensively studied over the past 30 years. Research in this area has looked for ways to increase the wear resistance of solid lubricant coatings at different contact loads and over a relatively wide range of temperatures and air humidity. Moreover, researchers have sought to find fundamentally new forms/structural states that can lead to new properties [11,12,13,14]. The testing temperature range has been, as a rule, between room temperature and ~500 °C. In most cases, coatings were applied through ion (magnetron) sputtering [10,11,12]. The structural and chemical state properties of these coatings have been achieved through pulsed laser deposition (PLD) [15,16,17] and liquid-based electrodeposition [18].

The most common structural state of deposited coatings is “amorphous”, which is generally assumed to form from nanoclusters with a lamellar structure typical of the 2H-MoS_2_ phase [19,20,21,22]. Friction sliding is associated with the tribo-induced crystallization of such coatings. During this process, a tribo-layer with enhanced lamellar structure ordering forms on the coating surface in the friction zone [22]. Most studies that have obtained Mo/W/S*_x_*/Se*_x_* coatings have done so with a chalcogen-to-metal ratio within ~1 < *x* ≤ 2, and have tested these coatings for tribological properties. When the mentioned ratio declines, the coefficient of friction either decreases or grows as the composition of the coatings approaches stoichiometric values [23,24]. The nature of this dependence is affected to some degree by the structural state of the coatings (the texture).

In general, an MoS*_x_* material with an amorphous structure that is obtained through physical vapor deposition and chemical synthesis has an S/Mo element ratio within a broader range (~1 ≤ *x* ≤ 10) [25,26]. The concentration of S atoms affects the formation of local atomic packing into the cluster units that form the MoS*_x_* amorphous compounds [26,27,28]. In the Supplementary Materials (Figure S1), we consider the possible variants for atomic packing and chemical bond organization in such nanoclusters. One of the main Mo_3_–S-type nanostructures consists of clusters (Mo_3_S_6_, Mo_3_S_9_, Mo_3_S_13_, and Mo_3_S_12_), where three Mo atoms form a triangle and are held in this configuration by chemical bonds with S atoms located between them [27,28,29,30]. When the concentration of S atoms changes, the chemical bond organization may change both within and between Mo_3_–S clusters.

To study atomic packaging in materials with an MoS_3_ composition, the Mo–S_3_ (-Mo-3S-Mo-3S-) linear chain model (Hibble et al. [31,32]) was employed alongside the model outlined above (Weber et al. [30]). The structural element of this model is an MoS_6_ cluster, where a Mo atom is surrounded by 6 S atoms. S^2−^ and S_2_^2−^ ligands mold the Mo atoms into a curved chain. MoS_3_-based nanomaterials may exhibit better antifriction behavior at normal temperatures and humidity when they are in the form of coatings or additives (to hydrocarbon lubricants) [33,34,35]. The tribo-induced formation of a tribo-film with a 2H-MoS_2_ lamellar structure was found on the contact surface of these nanomaterials in normal environmental conditions (in terms of sliding friction). The tribological properties of MoS_3_ and other MoS*_x_*_>3_ coatings have not been sufficiently studied in low-temperature adverse conditions. 

In the present work, we studied the possibility of applying laser-based processes to obtain sufficiently smooth and uniform low-friction nanocoatings with different sulfur contents (MoS_2_, MoS_3_, and MoS_4_). We determined the optimal structural and chemical states of clustered-type MoS*_x_* coatings, i.e., with the lowest coefficient of friction and most resistance to wear during sliding friction in an oxidizing environment at low temperatures. To prepare the thin-film coatings, a technique involving reactive pulsed laser deposition (RPLD) of laser-ablated plumes from the Mo target in reactive H_2_S gas was developed. Special deposition conditions (substrate temperature, laser fluence, hydrogen sulfide pressure, and distance from the substrate to the target) were selected to ensure that the growing coatings were bombarded by laser plasma ions. Ion bombardment of the growing coatings facilitated the formation of sufficiently dense amorphous structures with good adhesion to the steel substrate.

Analyses of the structure and chemical states of the elements in the obtained RPLD MoS*_x_* coatings (2 ≤ *x* ≤ 4) showed that sulfur content had an important effect on the local packing of atoms in the clustered-type structures of these coatings. An increase in sulfur content facilitated the transformation of the clustered-type structure in such a way that the combination of layered MoS_2_ clusters with weakly ordered Mo–S_3_/Mo_3_–S clusters was replaced by a polymer-like structure consisting of Mo_3_S_12_/Mo_3_S_13_ cluster units. It was apparent that the type of cluster and the characteristics of atomic packing had an important effect on the tribological properties of amorphous MoS*_x_* coatings at a low temperature in an oxidizing medium.

Comparative analyses of MoS_2_, MoS_3_, and MoS_4_ thin-film coatings showed that the best antifriction properties in adverse sliding friction conditions (a temperature of −100 °C and an environment containing water and oxygen molecules) were observed for MoS_3_ films composed of disordered Mo–S_3_/Mo_3_–S cluster units. In that case, the wear of the counterbody (a steel ball) when it slid over the surface of the MoS_3_ coating was at its lowest. During the sliding of the counterbody over MoS_2_ films containing the layered MoS_2_ cluster units, the removal of material from the wear track was minimal; however, the coefficient of friction and the wear of the counterbody were significantly higher than with MoS_3_ films. The tribological properties of MoS_4_ coatings that contained locally ordered Mo_3_S_12_/Mo_3_S_13_ cluster units were unsatisfactory. To explain these differences in the tribological properties of the coatings, we studied the tribo-induced modification of clustered-type coatings. Furthermore, we analyzed the mechanisms of structure modification due to tribo-impact and exposure to oxygen and water; these influences and their impacts on the tribological characteristics of the coatings were discussed.

It is important to note that homogeneous/single-layer MoS*_x_* coatings have a narrow scope, since they do not withstand a high load. Therefore, it is important to create nanolayered or nanocomposite coatings that contain an MoS*_x_* nanophase with enhanced tribo-characteristics. Our work thus focused on the study of single-layer MoS*_x_* coatings in order to then proceed to create MoS*_x_*-containing coatings with a more complex architecture. Previous studies have shown that nanolayered coatings containing alternating ultrathin MoS*_x_* layers (with an optimal concentration of sulfur) and diamond-like carbon (DLC) layers can exhibit a higher tribological performance during friction at a temperature of −100 °C in an oxidizing environment (compared to single-layer MoS*_x_* or DLC coatings). In our study, nanolayered coatings which contained an MoS_3_ solid lubricant demonstrated the best antifriction properties in complicated conditions.

## 2. Materials and Methods 

Nd/YAG (yttrium aluminum garnet) laser radiation at a wavelength of 1064 nm was used for the ablation of the Mo target. The duration of laser pulses was 15 ns, with an energy of ~85 mJ. The laser pulse frequency was 25 Hz. A laser fluence of ~20 J/cm^2^ was chosen to minimize droplet formation during the pulsed laser ablation of the Mo target. A standard configuration of reactive pulsed laser deposition was used to obtain the MoS*_x_* thin-film coatings. The angle between the laser beam and the target surface was ~45°. The substrate was placed parallel to the target surface, and thus, perpendicular to the laser plume flow. The distance between the Mo target and the substrate was ~3.5 cm. The substrate was kept at room temperature. The deposition chamber was evacuated to a pressure of 10^−2^ Pa. Then, H_2_S was injected into the deposition chamber. Film deposition was conducted at three different H_2_S pressures. In accordance with preliminary studies by Fominski et al. [36], H_2_S gas pressures of 8, 16, and 29 Pa were selected. The deposition time of the films was 20 min.

The laser plasma generated upon the irradiation of the Mo target was studied through time-of-flight spectrometry. To collect the time-of-flight spectra of Mo ions, a Langmuir plasma probe was installed 3.5 cm away from the Mo target. A probe diameter of 0.8 cm was maintained at a bias of –40 V. This bias voltage was sufficient to reject all plasma electrons and obtain a pure ion signal. The collected ion signals were used to assess the Mo ion fluxes that could influence the film growth. 

The deposition substrate was polished on 95Cr18 stainless steel discs (a C content of 0.95% and a Cr content of 18%). To determine the thicknesses of the MoS*_x_* films, the substrate was pressed tightly against the shield, which was removed after coating deposition. Then the thickness was measured using optical profilometry.

The surface morphologies of MoS*_x_* coatings and their chemical compositions were studied by scanning electron microscopy and by energy-dispersive X-ray spectroscopy (SEM/EDS, Tescan LYRA 3) both before and after tribo-testing. To determine the S/Mo atom content ratio, thin MoS*_x_* films were deposited on the polished Si substrates for 2 min. These samples were studied using Rutherford backscatter spectroscopy (RBS) on He ions (an ion energy of 1.5 MeV, a scattering angle of 160°, and an ion beam diameter of 100 μm). Mathematical modeling of the RBS spectra was performed using the SIMNRA program. The crystal structure of the coatings deposited on the steel substrates was examined using grazing incidence (10°) X-ray diffraction (XRD) with Cu Kα radiation (λ = 0.15406 nm) in an Ultima IV (Rigaku) diffractometer. The chemical states of the MoS*_x_* coatings were studied by X-ray photoelectron spectroscopy (XPS). XPS spectra were obtained by a Theta Probe Thermo Fisher Scientific spectrometer with a monochromatic Al Kα X-ray source (1486.7 eV) and an X-ray spot size of 400 μm. The photoelectrons had a take-off angle of 50° with respect to the surface plane. The spectrometer energy scale was calibrated using Au4f_7/2_ core level lines with a binding energy of 84.0 eV. The structure of the coatings before and after tribo-testing was studied by means of micro-Raman spectroscopy (MRS) using a 632.8-nm (He–Ne) laser, where the laser beam cross-section was less than 1 μm. The modes of the MRS spectrum measurements (laser beam intensity, measurement time) were selected in such a way that structural and chemical changes would not occur in the MoS*_x_* coatings.

To study the structure of MoS*_x_* coatings at the nanoscale level, thin MoS*_x_* films were deposited on NaCl substrates. The conditions for obtaining the thin MoS*_x_* films were the same as those used for producing the coatings on steel discs. The thin films were studied using transmission electron microscopy (TEM, including high-resolution TEM) and selected area diffraction (SAED) in a JEM-2100 (200 keV, JEOL, Japan) microscope. The samples were submerged in water and later the films were transferred to the microscope to produce a planar image. 

The tribo-testing of the thin-film coatings was carried out on an Anton Paar TRB3 tribometer in reciprocating motion mode, using a steel ball (100Cr6) with a diameter of 6 mm as a counterbody. The load on the ball was 1 N, and the Hertzian contact stress was 660 MPa. The average speed of the ball over a substrate with an MoS*_x_* coating was 1 cm/s. The length of the wear track was 5 mm. The tested steel discs were mounted in a holder cooled with liquid nitrogen. To prevent water vapor condensation and ice layer formation, friction tests were conducted in a mixture of air and argon. The tribometer, which was modified by the authors, is shown in Figure S2. An optimal argon flow rate allowed us to keep the disc temperature at −100 °C and to control the partial pressure of the air in the gas mixture surrounding the tested sample. The relative humidity (RH) of the air–argon mixture around the tested sample did not exceed RH ≤ 9%. For comparison, tribo-tests were performed at 22 °C in an air–argon mixture (RH ≤ 9%) and in air at RH ~ 50%. Four-hundred cycles of tribo-tests were conducted. The duration of testing revealed the key friction and wear properties of the obtained thin-film MoS*_x_* coatings.

Characterization of the wear tracks on the coatings, the wear scars on the balls, and the wear debris was carried out using a Wyko optical profilometer and optical microscopy. The tests were performed both before and after ultrasonic cleaning of the disk surface. The cleaning made it possible to remove low-adhesion wear particles. After tribo-testing, additional studies of the samples were carried out using SEM, EDS, and MRS techniques.

## 3. Results

### 3.1. The Structure and Composition of MoS_x_ Films

**Figure 1:**
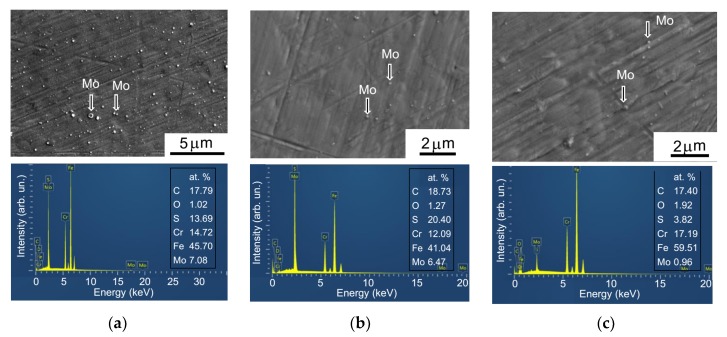
SEM images and energy-dispersive X-ray spectroscopy (EDS) spectra of MoS*_x_* thin-film coatings, obtained through reactive pulsed laser deposition (RPLD) on steel disks in H_2_S gas at pressures of (**a**) 8 Pa, (**b**) 16 Pa, and (**c**) 29 Pa. EDS spectra were measured in a 10 × 10 μm^2^ area. The arrows indicate the Mo particles that were ejected from the Mo target during pulsed laser ablation.

Figure 1 shows SEM images of the MoS*_x_* thin-film coatings obtained by RPLD at different pressures of the reactive H_2_S gas. The coatings had a rather smooth surface and a dense structure. No granular morphology, which is typical of MoS*_x_* ≥ 3 films obtained through reactive magnetron sputtering, was detected [28]. Individual submicron rounded particles were visible on the surface of the coatings. The appearance of these particles, which consisted of pure Mo, was due to the pulsed laser ablation of refractory metal. Laser ablation is always accompanied by the explosive boiling of molten metal, which causes submicron droplets (liquid phase) to splash. These particles escape the target at a very high rate and are likely to adhere to the substrate during deposition. At high temperatures, hot Mo particles do not have enough time to become saturated with sulfur. They are not transformed into an MoS_2_ compound on the substrate, either in the movement phase or in the cooling phase.

EDS studies showed that the S/Mo ratio for MoS*_x_* coatings obtained at an H_2_S pressure of 8 Pa was *x* ~ 2. When the H_2_S pressure was increased to 16 Pa, the relative sulfur content was *x* ~ 3. At a pressure of 29 Pa, it was *x* ~ 4. A chemical mapping of the Mo, S, and Fe distributions over the surface of the covered steel substrate indicated the uniform surface deposition of Mo from the laser plume (Figure S3). The physical vapor deposition of Mo atoms was assisted by uniform chemical vapor sulfur deposition from the H_2_S gas. The relative EDS intensity of the peaks from substrate elements decreased slightly as the pressure increased from 8 to 16 Pa, but grew markedly when the MoS*_x_* coatings were deposited at 29 Pa. The profilometric measurements of the MoS*_x_* film thicknesses showed that the thickness of the MoS_2_ coatings was approximately 500 nm. An increase in the H_2_S pressure caused the thickness to decrease to ~410 nm in the MoS_3_ coatings and to ~300 nm in the MoS_4_ coatings. It is well known that the growth rate of coatings during the PLD process decreases in a buffer gas medium due to the scattering of laser plume atoms through collisions with gas molecules. The efficiency of the scattering process increases with increasing gas pressure, which causes a deviation in the trajectory of the atoms at large solid angles; as a result, there is an increase in the laser plume deposition area. In the case of RPLD of MoS*_x_* coatings through Mo deposition in a reactive H_2_S gas medium, the loss of deposition in Mo atoms from the laser plume is compensated for, to some extent, by the adherence of active S atoms from H_2_S gas, which is activated due to interactions with the laser plasma from the Mo targets.

Considering that the Mo Lα and S Kα peaks overlapped, and that EDS cannot unambiguously demonstrate the stoichiometry of MoS*_x_* coatings, we also measured the S/Mo ratio through RBS. The RBS spectra for the thin MoS*_x_* films on the Si substrate are shown in Figure S4. SIMNRA modeling of the experimental spectra allowed us to estimate that the atomic ratio *x* increased from *x* ~ 1.9 ± 0.1 to *x* ~ 4.3 ± 0.2 when the H_2_S pressure increased from 9 to 29 Pa. At a pressure of 18 Pa, the ratio was *x* ~ 3.2 ± 0.1. The S/Mo ratio results from the RBS and EDS measurements had satisfactory agreement.

The measurements of the ion fluxes that bombarded the MoS*_x_* coatings during RPLD growth indicated the following (Figure S5). The ion bombardment dose decreased with increasing H_2_S pressure. However, the growth rate of these coatings also decreased at the same time. Within the applied H_2_S pressure range, the coating growth rate was correlated with the ion bombardment dose. The form of the ion pulses did not change significantly as the pressure grew. This means that, overall, the energy distribution of the ions affecting the formation of the coating structure stayed the same. This circumstance may explain the formation of a rather dense structure in the deposited MoS*_x_* coatings. If the ion bombardment of MoS*_x_* coatings (which are formed through the deposition of Mo and S atoms) is absent or ineffective, there is a tendency toward a porous column structure with a cauliflower morphology [37]. 

**Figure 2:**
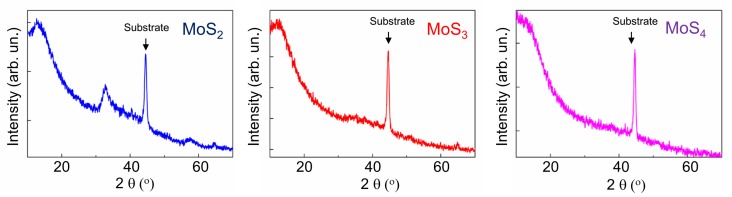
XRD patterns for different MoS*_x_* films obtained through RPLD in H_2_S gas on the steel substrates.

Figure 2 shows the results of the XRD studies of MoS*_x_* coatings. The intensive and narrow peak at 2θ ~ 44.5° was due to reflection from the steel substrate. The diffractograms of the MoS_2_ coatings were similar in many respects to those measured for transition metal–dichalcogenide-based coatings obtained through ion sputtering or PLD at room temperature [19,21,22,38]. The XRD patterns of these coatings showed an intense and broad peak near 2θ ~ 13° and a pronounced peak at 2θ ~ 33°, followed by a broad peak at 2θ ~ 43°. The latter had a long tail toward higher 2θ values and was highly asymmetric. These peaks corresponded to the X-ray diffraction results from the basal (002) and (100) planes and turbo-straight stacking of (10 *L*) planes (*L* = 1, 2, 3) in the nanostructured 2H-MoS_2_ phase. According to the model proposed by Weise et al. [19], such XRD patterns can be explained by the two-dimensional (2D) organization of the basal planes, which can be several tenths the size of the unit cell dimensions. As the lateral dimensions of the basal planes decreased, either a broadening or a drop in the intensity of the (10 *L*) planes occurred until the low intensity and broad peak typical of an amorphous structure were detected. 

Broad and featureless diffraction peaks were observed in the XRD patterns of the MoS_3_ and MoS_4_ films obtained through RPLD (Figure 2). This meant that local atomic packing in the MoS*_x_* films with an increased sulfur content could differ significantly from the atomic packing that was typical of the basal planes of the 2H-MoS_2_ phase. The X-ray diffractogram of the RPLD MoS_3_ coatings was very similar to that of amorphous MoS_3_ material obtained through chemical synthesis, e.g., through facile wet chemistry [27] or reactive magnetron sputtering [28]. 

**Figure 3:**
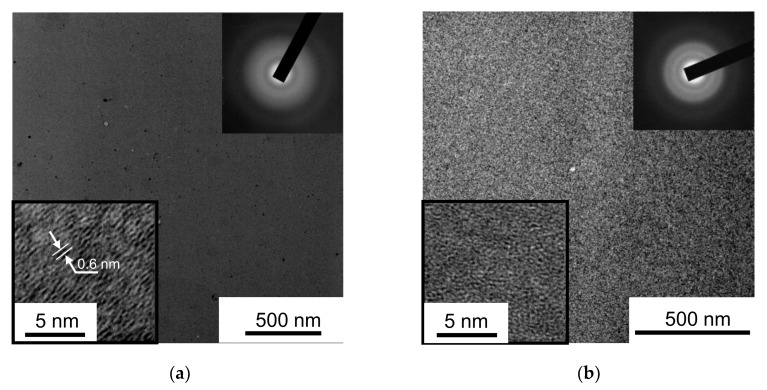
TEM images and selected area diffraction (SAED) patterns of the thin films obtained through RPLD: (**a**) MoS_2_ and (**b**) MoS_3_. The bottom inserts show high-resolution TEM images of the films.

TEM image contrasts and SAED patterns confirmed the amorphous and quite homogeneous structure of the MoS*_x_* films obtained through RPLD (Figure 3 and Figure S6). The TEM images had a featureless contrast, and the SAED patterns contained several diffuse–broaden intensity maxima for all of the analyzed areas of the samples. In the MoS_2_ films, a comparison of the SAED patterns to the X-ray diffraction results (Figure 2) showed that in both diffraction patterns, there was a noticeable maximum with a wavenumber of ~11 nm^−1^, as well as a relatively pronounced diffuse maximum with a wavenumber of 23.6 nm^−1^. This analysis of XRD data indicated the possibility of local organization of Mo and S atoms during film growth, which caused the formation of MoS_2_ nanoclusters with laminar packing in the basal planes. The high-resolution TEM image contrasts of these films had nanosized, mutually oriented/parallel lattice fringes, which confirmed the formation of layered-type MoS_2_ nanocrystals. The characteristic distance between the planes and the average number of stacked planes were ~0.6 nm and ~4, respectively. The characteristic length of the nanocrystals did not exceed 4 nm. The layered nanocrystals were incorporated into the amorphous matrix of the MoS_2_ film.

In the MoS_3_ films, the electron diffraction pattern contained a maximum of intensity with a small wavenumber (~11 nm^−1^), as did the X-ray diffraction pattern. This indicated the possibility of clusters forming with layered packing, which is characteristic of MoS_2_. However, the TEM images of these films consisted mainly of randomly oriented threads. It was possible to distinguish very small areas (~2 nm) of images in which local ordering and layered packing were observed. The SAED pattern of the MoS_3_ films also contained broadened and relatively intense diffraction rings corresponding to wavenumbers of ~23.6 and ~34.5 nm^−1^. The broadened peaks, which corresponded to the same wavenumbers but were characterized by relatively low intensities, were also detected when measuring the X-ray diffraction patterns of the MoS_3_ coatings (Figure 2). The wavenumbers of the diffraction maxima for amorphous MoS_x_ films were determined either by the distribution of atoms in the coordination spheres (in a completely homogeneous amorphous material) or by the superposition of diffraction maxima from local regions of the films, which contained different cluster structures. For the Mo‒S_3_ and Mo_3_‒S clusters (Figure S1), the distances between the nearest Mo–Mo and Mo–S atoms were ~3–3.2 and 2–2.1 nm, respectively. Thus, the first diffraction maxima from the first coordination sphere could occur with wavenumbers of 24 and/or 36.8 nm^−1^. These data coincided quite well with the experimental data from the SAED of the MoS_3_ films.

The structure of the MoS_4_ films was not stable enough to study it using TEM, as under electron beam irradiation, the structure of MoS_4_ films can change. The results from the TEM/SAED studies of these films are shown in Figure S6. The structure could have been modified due to the removal of sulfur from the films and the formation of nanocrystals enriched in molybdenum (for example, Mo_2_S_3_).

**Figure 4:**
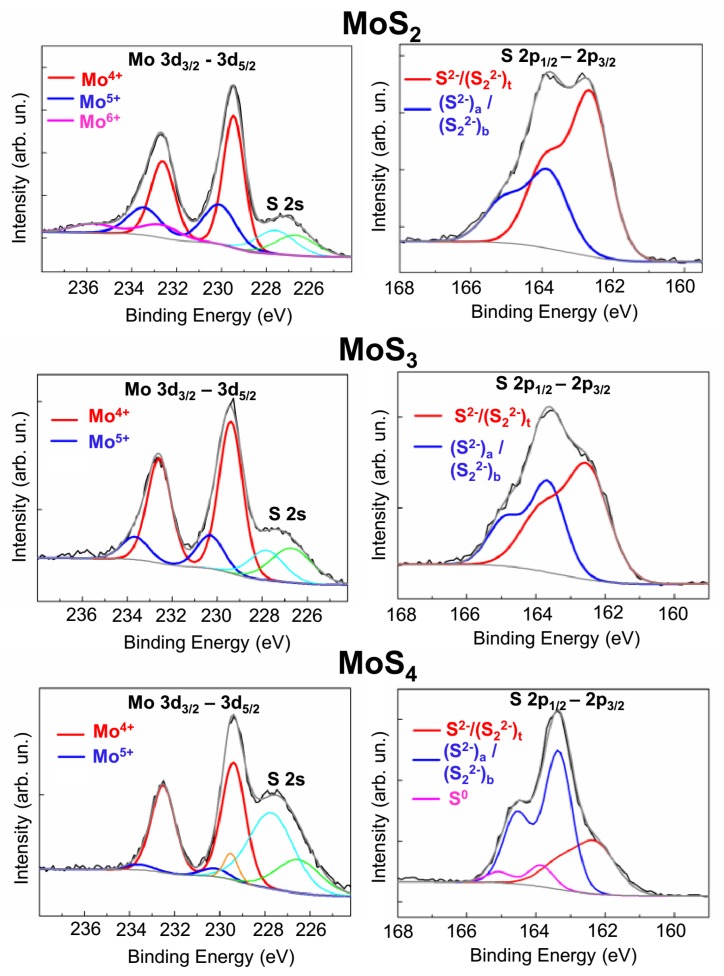
XPS spectra of Mo 3d and S 2s, measured on the surfaces of MoS_2_, MoS_3_, and MoS_4_ coatings.

XPS studies showed substantial differences in the chemical states of the MoS_2_, MoS_3_, and MoS_4_ coatings (Figure 4). For the MoS_2_ coating, three Mo 3d_5/2_–3d_3/2_ doublets (attributable to three valences of molybdenum) were present in the XPS Mo 3d spectrum. The peak positions of Mo 3d_5/2_ and 3d_3/2_, which were at 229.4 eV and 232.5 eV, respectively, were indicative of Mo^4+^. Mo 3d_5/2_–3d_3/2_ doublets are characteristic of Mo^5+^ and Mo^6+^ when the peaks are at 230.2–233.3 eV and 232.7–235.8 eV, respectively. Mo^4+^ states have previously been observed in lamellar 2H-MoS_2_ compounds and in amorphous compounds based on Mo_3_–S clusters [27,39,40]. An Mo^5+^ state is more typical of materials based on Mo–S_3_ clusters [33,34,40]. An Mo^6+^ state is caused by the formation of molybdenum oxides. These states could have occurred after the coating was exposed to air when it was removed from the vacuum chamber. Probably, the surface layer of the MoS_2_ coating, which was obtained at low H_2_S gas pressures, retained the Mo atoms that did not have a chemical reaction with sulfur. When the sample was removed from the deposition chamber, these atoms had a chemical reaction with O-containing air molecules.

Remarkably, the Mo 3d spectrum overlapped with the S 2s spectrum. The S 2s spectrum was split into the following singlets: a singlet with a binding energy of 226.4 eV, attributable to the S^2−^ states in MoS_2_; a singlet with a binding energy of 227.7 eV, with S_2_^2−^ and/or apical S^2−^ in Mo–S_3_ clusters with an increased sulfur content; and a singlet with a binding energy of 229.5 eV, with S^0^ polysulfide clusters. The Supplementary Materials (Figure S1) contain further details on the possible states of sulfur atoms in different clusters in amorphous MoS*_x_*.

An analysis of the chemical states of S atoms showed that, in the MoS_2_ coatings, the Mo^4+^ state was due to the formation of bonds with S atoms in layered-type MoS_2_ clusters. The S 2p spectrum was dominated by S 2p_3/2_–2p_1/2_ doublets with a low binding energy (162.3 and 163.5 eV for S 2p_3/2_ and S 2p_1/2_ peaks, respectively). This doublet is typical of the state of S atoms in crystal 2H-MoS_2_. The presence of a high-binding-energy doublet, in which S 2p_3/2_ and S 2p_1/2_ peaks had an energy of 163.4 and 164.6 eV, respectively, pointed to the formation of Mo_3_–S and Mo–S_3_ clusters. A sulfur doublet with a high binding energy is associated with, as a rule, bridging S_2_^2−^, shared S_2_^2−^, and/or apical S^2−^ [27,28,29,30]. The intensity of a doublet with a low binding energy was noticeably dominant in the S 2p spectrum of the MoS_2_ film, which indicated the predominance of layered-type MoS_2_ clusters in the amorphous structure of the MoS_2_ coatings. 

In the MoS_3_ coatings, there were Mo 3d_5/2_–3d_3/2_ doublets in the XPS spectrum of Mo 3d, which were attributable to Mo^4+^ and/or Mo^5+^ only. In the XPS spectrum of S 2p, the intensity of the doublets with a high binding energy significantly increased (Figure 4); this means that the proportion of Mo_3_–S clusters increased in the amorphous structure of the MoS_3_ coatings. In these clusters, sulfur ligands with an S 2p_3/2_ peak at 162.3 eV could be assigned to terminal S_2_^2−^ units or unsaturated S^2−^. The intensity of the low-binding-energy doublet was higher than that of the high-binding-energy doublet, which meant that the structure of the Mo_3_–S cluster was not perfect and that the amorphous structure had sulfur ligands causing bonding, turning Mo atoms into MoS_2_. The presence of Mo^5+^ pointed to the possibility of the local formation of Mo–S_3_ clusters. In the MoS_4_ coatings, the Mo 3d spectrum was approximated well through a doublet attributable to Mo^4+^. The contribution of doublets attributable to other Mo valences was insignificant. The S 2p spectrum was clearly dominated by high-binding-energy doublets. These factors pointed to the formation of a polymer-like structure consisting mainly of Mo_3_S_13_ and/or Mo_3_S_12_ clusters. These clusters differed only in that the Mo_3_S_12_ cluster lacked an apical S^2−^ ligand. In the S 2p spectrum, there was a doublet with an S 2p_3/2_ peak with a binding energy of nearly 163.8 eV. In MoS_4_ films produced through RPLD at the highest H_2_S pressure, this peak could have been due to the adsorption of H_2_S or other S-containing molecules formed by the interaction between laser plasma and the background gas on the surface of the growing coating. At a high rate of deposition of such molecules, the formation of polysulfide S^0^ clusters would have been possible [25,36].

### 3.2. Tribological Properties of the MoS_x_ Thin-film Coatings

**Figure 5:**
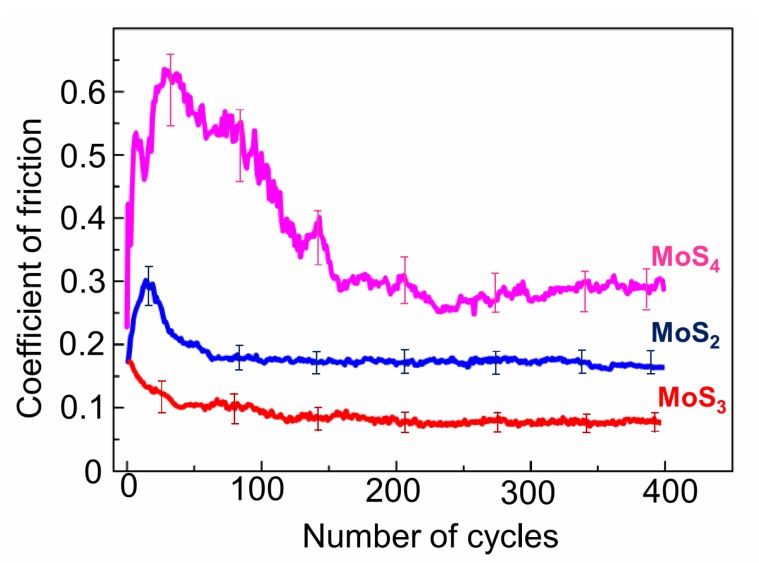
Characteristic evolution of the friction coefficient as a function of the cycle number (for MoS*_x_* coatings with different numbers of S atoms). Pin-on-disk tribometer testing was conducted at −100 °C in an argon–air mixture (relative humidity RH ~ 9%). The vertical lines show the range of deviation of the friction coefficient during three tracks of tribological testing on the same sample.

Figure 5 shows the results from measurements of the coefficient of friction when testing MoS*_x_* coatings with different sulfur contents at a temperature of −100 °C. The MoS_3_ coatings had the best and most stable solid lubricant properties. The coefficient of friction was reduced from an initial value of 0.17 to 0.1 over 40 sliding cycles, and then gradually diminished to ~0.08. The MoS_2_ coating required a longer run-in period. The friction coefficient first increased from 0.17 to 0.3 over 15 cycles and then decreased to 0.18 over 50 cycles. Then, the coefficient of friction changed insignificantly to reach 0.16 by the end of the test. The antifriction properties of the MoS_4_ coating were clearly inferior to those of the coatings with a lower sulfur content. The run-in period of the MoS_4_ coating exceeded 200 cycles. During that time, the friction coefficient increased to 0.62 and then decreased to 0.26. The coefficient of friction changed insignificantly during further tribo-testing.

**Figure 6:**
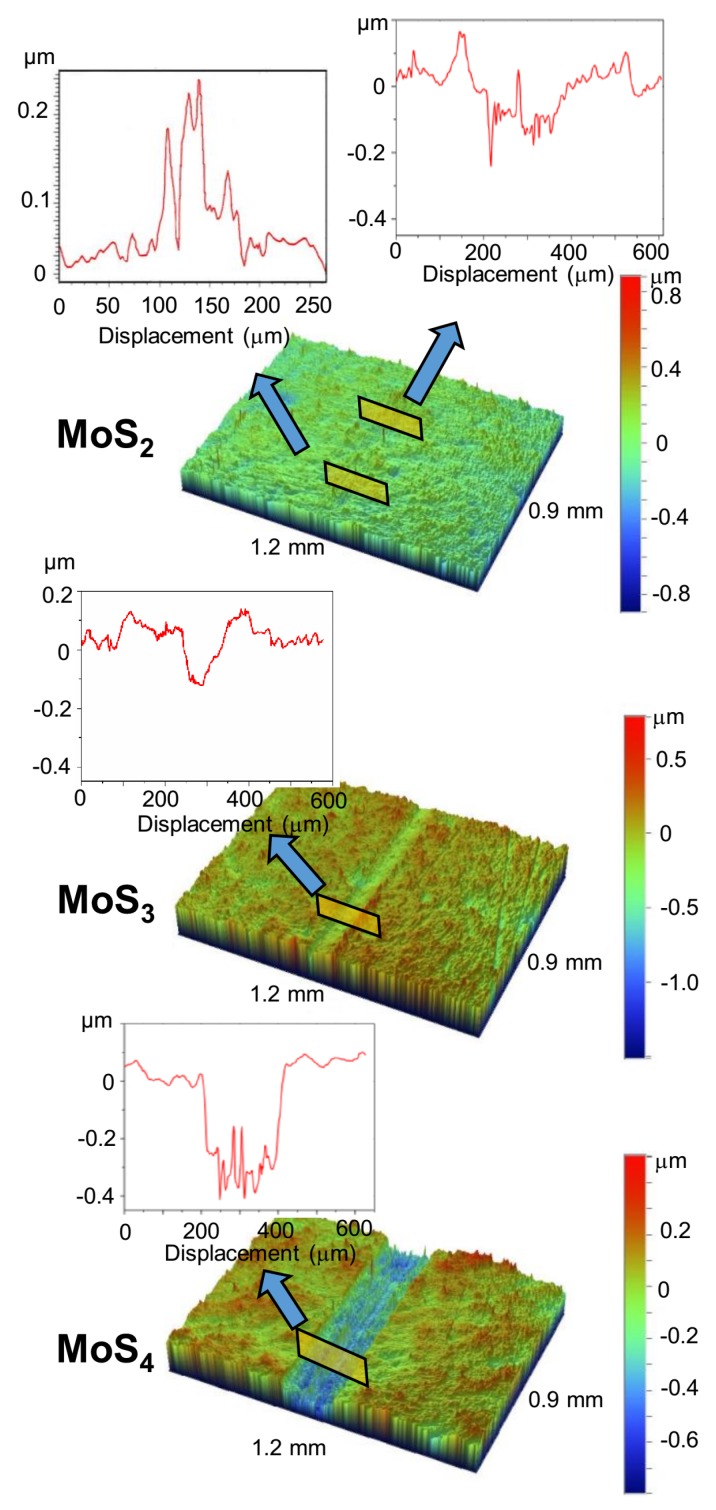
3D images of the central part of wear tracks formed on the surface of MoS_x_ coatings (with different S contents) after tribo-testing at −100 °C in an argon–air mixture (RH ~ 9%). The inserts illustrate the depth profiles of the wear scars on the coatings.

Figure 6 shows the results of the study of the wear tracks that formed after the tribo-testing of MoS*_x_* coatings. The profilometry of the central part of the wear track on the MoS_2_ coatings demonstrated uneven wear. A 150–200 nm deep groove formed in some local regions of the wear track. The width of the wear track was ~200 µm. In other regions of this track, protrusions were observed that were up to 200 nm high. The wear on the MoS_3_ coatings was more uniform throughout the track; as a result, a groove with a depth of 100–120 nm and a width of ~120 µm appeared. The MoS_4_ coating was subject to the most severe wear throughout the sliding track. The groove reached a depth of ~400 nm and a width of ~200 μm. A cross-sectional profile of the wear track indicated that, at the end of the test, the ball could touch the surface of the steel substrate.

It should be noted that the relative sliding velocity of the tribo-pair was the highest in the central part of the track, and it decreased when the ball approached the edge of the track. The tribo-pair experienced static friction at both edges of the track, which could have caused sticking in the pair. Profilometric studies showed that the wear on the MoS*_x_* coatings was fairly uniform along the entire length of the track. The results of these studies are shown in Figure S7. These results indicate that for the selected mode of ball sliding, the wear of the coatings did not significantly depend on the ball sliding speed. The effect of tribo-induced mass transport over the track surface, which leveled the depth of the tracks, cannot be excluded. A calculation of the volumes of MoS_x_ removed from the tracks during the sliding friction tests showed that these volumes were 0.8 × 10^5^, 1 × 10^5^, and 4.5 × 10^5^ μm^3^, respectively, for the MoS_2_, MoS_3_, and MoS_4_ coatings. For the MoS_2_ coatings, the areas with adhered wear debris were not considered in the wear calculations. 

**Figure 7:**
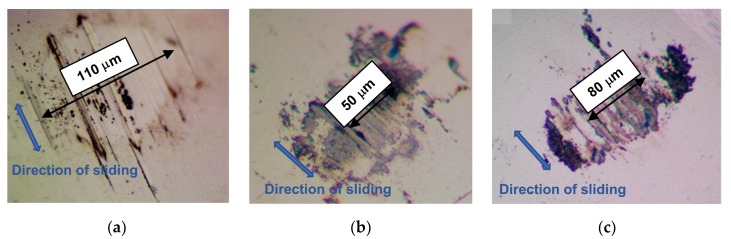
Optical images of wear scars formed on the steel balls after sliding against the (**a**) MoS_2_, (**b**) MoS_3_, and (**c**) MoS_4_ coatings at −100 °C in the argon–air mixture (RH ~ 9%).

Figure 7 shows optical images of the ball wear scars, which formed after the balls slid over various MoS*_x_* coatings. The largest wear scar (~110 µm) was observed in a ball that slid over the MoS_2_ coating. The wear debris adhered weakly to the ball. Considering the findings of the wear track studies, this pointed to the effective adherence of wear debris to the track surface in the region where the counterbody slid over the MoS_2_ coating.

The size of the wear scar formed on the ball after it slid over the MoS_3_ coating was minimal (~50 µm). A noticeable adhesion of wear debris was observed on the ball at the edges of the contact area. An increase in the size of the contact area during sliding (over the MoS_3_ coating) caused the wear track on this coating to widen to 200 µm. The size of the wear scar formed on the ball after it slid over the MoS_4_ coating was ~80 µm. As with the MoS_3_ coating, the ball exhibited a noticeable adhesion of wear debris at the edges of the contact area. As a result, the width of the wear track on the MoS_4_ coating exceeded the size of the wear scar on the ball.

## 4. Discussion

Further studies of the wear tracks using EDS confirmed the differences in the wear mechanisms, which were characteristic of MoS_2_ and sulfur-rich MoS*_x_* coatings (see Supplementary Materials, Figures S8–S10). For the MoS_2_ coating, when the analyzing electron beam moved across the wear track, the measurements of the intensity of the Mo Lα and S Kα peaks did not reveal any noticeable change in the intensity either in the region where the counterbody slid or at the borders of the track. Thus, as it barely moved from the track, the coating material was not transferred to the boundaries during tribo-testing. For the MoS_3_ and MoS_4_ coatings, the intensity of the Mo Lα and S Kα peaks markedly decreased in the ball-sliding region and increased at the boundaries of the track. These changes can be explained by both the transfer of material from the track to its boundaries in the course of wear and by the deformation of coating material under pressure from the counterbody. 

**Figure 8:**
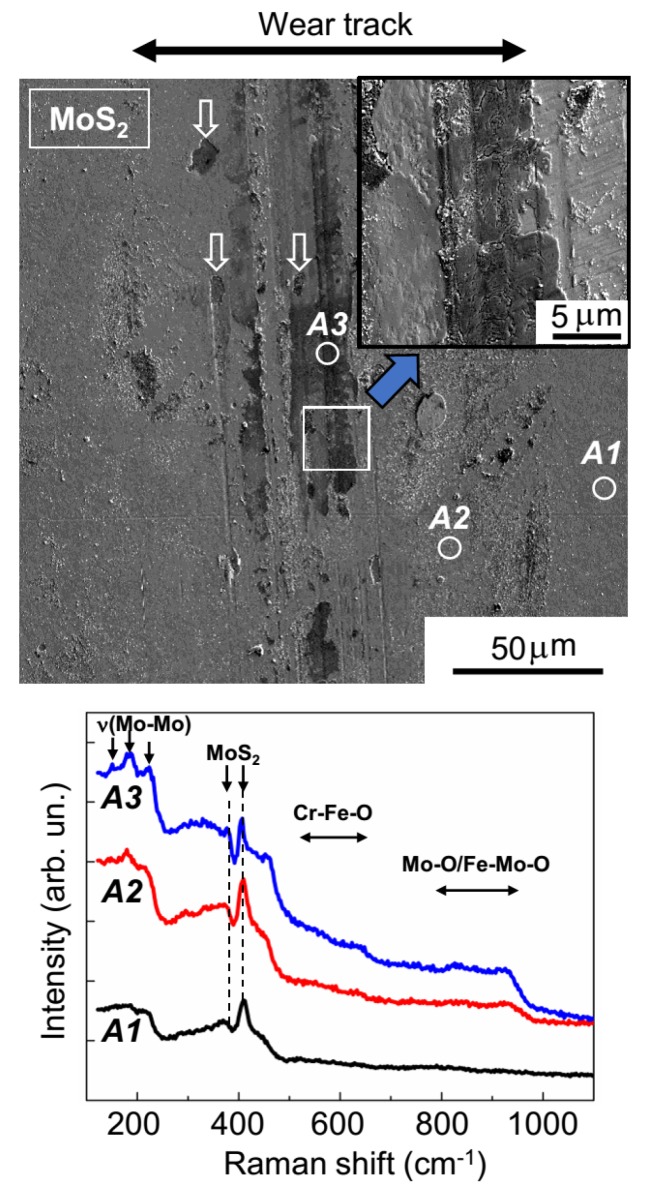
SEM image and Raman spectra for the MoS_2_ coating subjected to tribo-testing at −100 °C in the argon–air mixture (RH ~ 9%). Raman spectra were measured on the unworn surface of the coating (area *A1*) and on the wear track surface (areas *A2* and *A3*). In the figure, area *A3* shows the wear debris that adhered to the surface of the track and was then deformed by the ball. The white arrows indicate micrometer-sized areas of local delamination on the MoS_2_ coating.

The SEM results (shown in Figure 8) confirmed the uneven wear of the MoS_2_ coating. On the track surface, there were regions that had barely any wear, and there were also areas formed by the deformation of wear particles. In all track areas, the sliding of the counterbody activated the structural rearrangement of the MoS_2_ coating material. The MRS spectrum of the unworn coating (area *A1*, Figure 8) was attributable to its amorphous structure, in which there were regions of local atomic ordering into lamellar clusters (layer-type MoS_2_); this was evidenced by narrow peaks at 410 and 370 cm^−1^, which stood out clearly against the wide bands of the amorphous MoS_2_. These peaks were caused by the vibrations of A_1g_ and E_2g_^1^, respectively, in the hexagonal 2H-MoS_2_ structure [34,35,41]. For the MoS_2_ coating, in the regions of the sliding track that were not subject to substantial wear (area *A2*, Figure 8), the peak intensity grew significantly at 410 cm^−1^; this points to an increased content of lamellar material and/or increased crystallinity in these regions. During the activation of the development of the layered MoS_2_ structure, the sliding friction caused the formation of Mo–O and Fe–Mo–O compounds, which was evidenced by the emergence of a broadened band in the Raman spectrum, at 800–980 cm^−1^ [42,43]. An Fe–Mo–O compound (e.g., with Mo = O stretching in FeMoO_4_, the Raman shift was 925 cm^−1^) was probably formed during a reaction between molybdenum-based compounds and iron oxides on the surface of the steel ball. 

The formation of an Mo–O compound could have been due to the environment containing O_2_ and H_2_O on the MoS_2_ surface layer. In an orthorhombic a-MoO_3_ compound, the most intense peak in Raman spectra is located near 820 cm^−1^ (an O‒Mo_2_ stretching vibration) [44]. Changing the packing of the MoO_6_ octahedra (the main structural element of all polymorphic modifications of MoO_3_) may shift this line. For hexagonal h-MoO_3_, the strongest peaks are located between 830 and 930 cm^−1^ [45]. Although the h-MoO_3_ phase is metastable, when the ball comes into contact with the coating, local, nonequilibrium rapid heating and/or cooling of this material may occur due to friction. Therefore, the possibility that h-MoO_3_ nano-inclusions formed cannot be completely excluded. Furthermore, the condensation of H_2_O molecules can be accompanied by tribo-induced electrochemical corrosion. In this case, various Mo–O(–S) bonds can form whose vibration band range is between 800 to 1000 cm^−1^ [46].

The relative intensity of the peaks characteristic of Mo–O and Fe–Mo–O peaks significantly increased in the MRS spectrum. This was measured in the region where there was wear debris adhesion (area *A3*, Figure 8). In this spectrum, there were broadened peaks at 530–660 cm^−1^. One of the most probable causes of these peaks was the formation of an Fe–Cr–O compound [47]. The production of this compound in the track is easy to explain, given the relatively intense wear of the steel ball and the effective adhesion (and deformation) of wear debris on the MoS_2_ surface in the sliding track region. EDS studies confirmed the pronounced oxygen saturation of some local sections of the tracks that formed on the MoS_2_ coating after tribo-testing (Figure S8). The MRS spectra indicated an improvement in the quality of the local packing of MoS_2_ in these regions. This improvement manifested itself both in the narrowing of the main MoS_2_ peaks (at 370 and 410 cm^−1^) and in the appearance of additional peaks in the range of 150–225 cm^−1^ (the ν(Mo–Mo) vibration band) [46]. Wear debris with a high MoS_2_ content could have formed through local delamination of the MoS_2_ coating (indicated by arrows on the SEM images in Figure 8).

**Figure 9:**
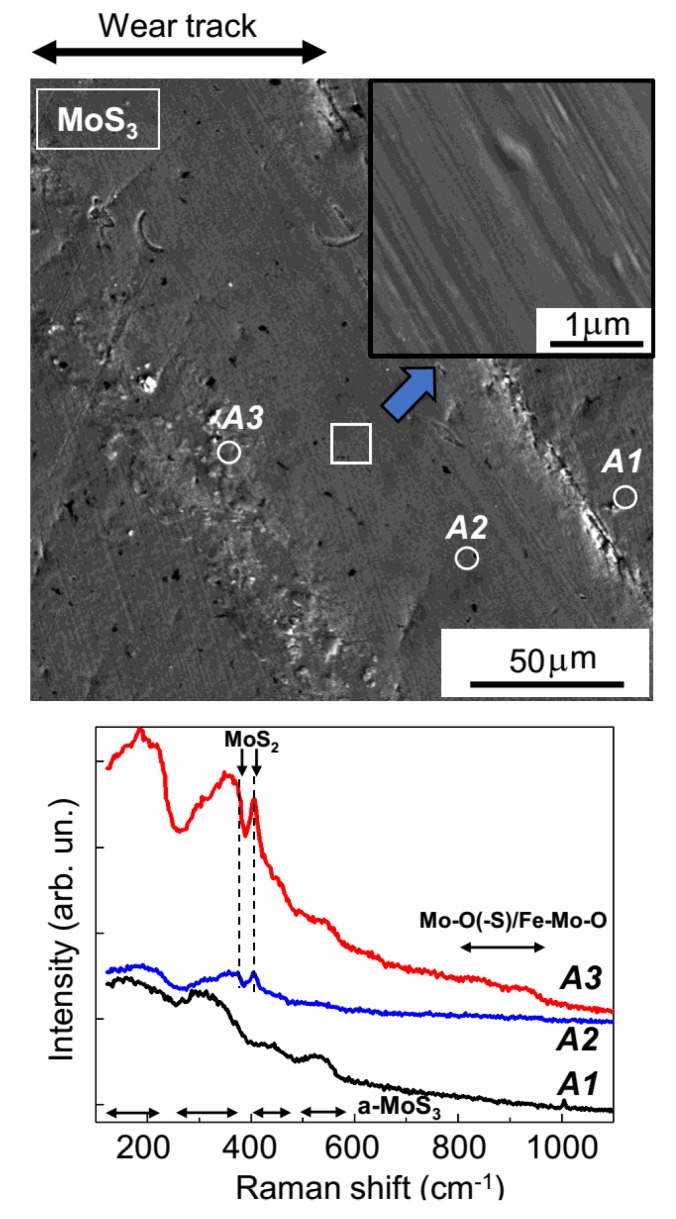
SEM image and Raman spectra of MoS_3_ coatings subjected to tribo-testing at −100 °C in the argon–air mixture (RH ~ 9%). Raman spectra were measured on the unworn surface of the coating (area *A1*), in the center of the wear track (areas *A2*), and at the track boundary, which contained adhered wear debris (area *A3*).

**Figure 10:**
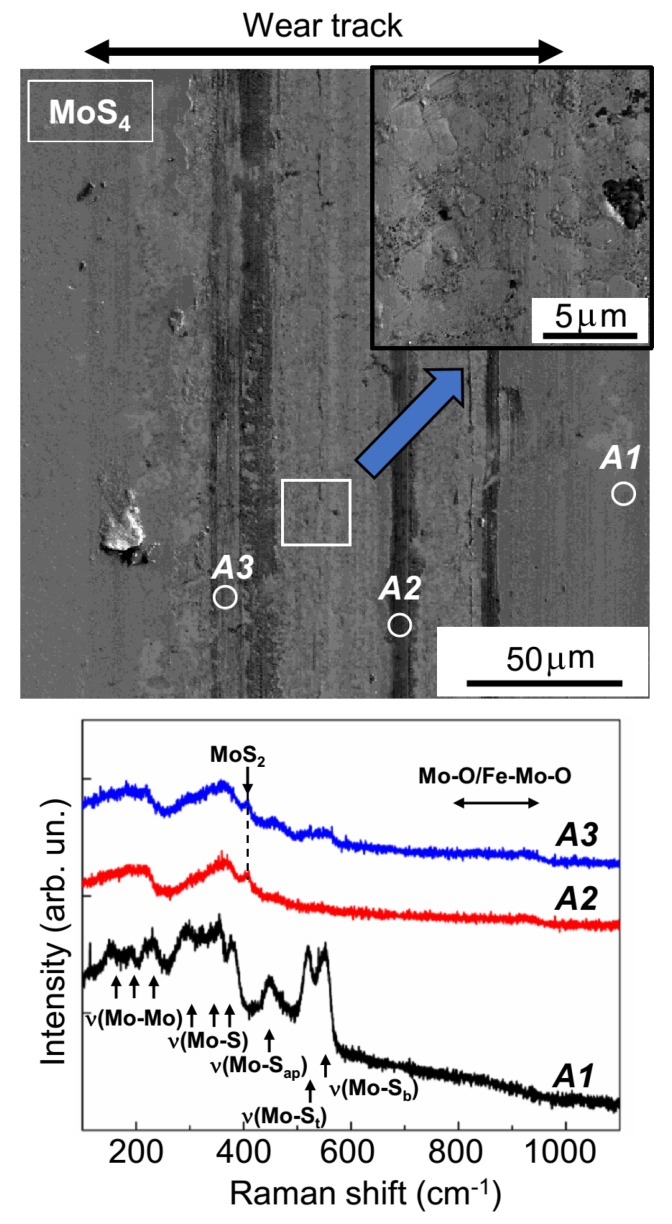
SEM image and Raman spectra of MoS_4_ coatings subjected to tribo-testing at −100 °C in the argon–air mixture (RH ~ 9%). Raman spectra were measured on the unworn surface of the coating (area *A1*) and in two different local areas of the wear track (areas *A2* and *A3*).

Figure 9 and Figure 10 show the results from the SEM and MRS studies of wear tracks on the surfaces of MoS_3_ and MoS_4_ coatings, respectively. The Raman spectrum of pristine MoS_3_ coatings (area *A1*, Figure 9) had four broad peaks at 200, 320, 450, and 540 cm^−1^. This spectrum is typical of amorphous MoS*_x_*_~3_ materials that lack qualitative ordering into clusters (Mo_3_S_12_/Mo_3_S_13_-type) [25,36,39]. These clusters have narrower peaks that occur at the following vibration modes: ν(Mo-Mo) at ~200 cm^−1^, ν(Mo-S)_coupled_ at ~320 cm^−1^, ν(Mo-S_apical_) at ~450 cm^−1^, ν(S-S)_terminal_ at ~520 cm^−1^, and ν(S-S)_bridging_ at 540 cm^−1^ [46]. The Supplementary Materials (Figure S1) provide further details on the various sulfur ligands in the clusters of the Mo_3_S_12_/Mo_3_S_13_-type. In unworn MoS_4_ coatings, a spectrum with these peaks as well as other additional peaks was observed (area *A1*, Figure 10). Thus, the MRS studies confirmed a significant difference in terms of local atomic packing in the MoS_2_, MoS_3_, and MoS_4_ coatings.

The central area of the wear track that formed on the MoS_3_ coating had a rather smooth surface, indicating the perfect ductility of the coating material. After tribo-testing, sharp peaks appeared at 370 and 410 cm^−1^ in the MRS spectrum (area *A2*, Figure 9) over a background of broad bands from the amorphous structure; this indicated the modification of the amorphous structure and the formation of layered-type MoS_2_ inclusions in the amorphous MoS_2_ phase. No noticeable peaks attributable to Mo–O and/or Fe–Mo–O compounds were visible in the MRS spectrum in the center of the track. Noticeable bands in a frequency range from 800 to 950 cm^−1^ were detected in the spectra measured at the track boundaries (e.g., area *A3*, Figure 9). In these areas, the intensity of peaks of the MoS_2_ phase increased significantly. Given the EDS results, which indicated an increase in the Mo, S, and O contents at the track boundaries (Figure S9), it should be assumed that during testing of the MoS_3_ coatings, wear particles containing both Mo–O/Fe–Mo–O compounds and sufficiently crystallized MoS_2_ nanoparticles accumulated at the track boundaries.

The SEM studies showed that the surfaces of the wear track on the MoS_4_ coatings were less smooth than they were on the MoS_3_ coatings (Figure 10). According to the Raman spectra shown in Figure 10, when sliding, the counterbody caused the formation of layered-type MoS_2_ clusters in all track sections, accompanied by the formation of Mo–O and/or Fe–Mo–O compounds (the band at 800–930 cm^−1^). The local packing typical of Mo_3_S_12_ and Mo_3_S_13_ moieties was completely distorted; this was evidenced by the disappearance of narrow peaks and the appearance of wide bands in the Raman spectra, which were measured in the region where the counterbody slid. EDS studies confirmed the relatively increased and fairly uniform distribution of oxygen atoms over the track surface on the MoS_4_ coatings (Figure S10).

Thus, the sliding friction of the steel counterbody at −100 °C on coatings with different sulfur contents caused the transformation of the amorphous structure and probably the formation of a tribo-film with a layered-type MoS_2_ structure. It is well-known that the formation of such tribo-films at the sliding interface ensures sufficient low-friction properties [34,41,48,49]. The coefficient of friction and the wear resistance of the MoS_2_, MoS_3_, and MoS_4_ coatings, however, differed significantly. All coatings had a rather dense structure. Thus, here it is difficult to accept the common explanation of the influence of an oxidizing environment on the tribological properties of MoS*_x_*-based coatings. One of the main reasons an oxidizing environment has an influence is the penetration of O-containing molecules into the coating through microcracks and structure defects [50]. It is unlikely the same influence exists in terms of sliding friction on MoS_2_, MoS_3_, and MoS_4_ coatings.

Another important factor affecting the tribological properties of MoS*_x_* coatings is the orientation of basal planes in the MoS_2_ lamellar structure with respect to the surface of the coating [49]. The orientation of reference planes can be seen in A_1g_ and E_2g_^1^ peak intensities in Raman spectra. In Raman spectra of terrace-terminated MoS_2_ films, which have the most qualitative tribological properties, the contribution of the E_2g_^1^ Raman mode increases. In Raman spectra of edge-terminated MoS_2_ films, the A_1g_ Raman mode may be more pronounced. In Raman spectra of most tribo-films, the peak at 370 cm^−1^, which is attributable to the E_2g_^1^ Raman mode, is expressed weakly; this could be due to signal overlap from resonance scattering on wear particles [41]. Here, the Raman spectra of the tribo-films formed on the MoS_4_ coatings had virtually no peak at 370 cm^−1^, which can be explained by the tribo-film’s specific texture, as the basal planes were oriented mainly perpendicularly to the surface of the coating.

MoS*_x_* structure modification and tribo-film formation can be influenced by the atmosphere during sliding friction, primarily by the concentration of O_2_ and H_2_O molecules. The role of these reagents can differ [51,52]. Additional tribo-testing of the MoS_2_, MoS_3_, and MoS_4_ thin-film coatings was carried out at room temperature and at varying atmospheric humidity. In the Supplementary Materials, Figures S11–S14 present the results of this testing. The MoS_4_ coatings showed unsatisfactory tribological properties under all tribo-testing conditions, which was evident in the high coefficient of friction and fast wear. These results indicate that atomic packing in an amorphous MoS_4_ structure could not ensure the effective tribological adaptation of the structure in all friction conditions. Probably, the layered-type MoS_2_ clusters detected did not form a continuous tribo-film, but rather, mixed with metal oxide clusters.

The coefficient of friction for the MoS_2_ coatings clearly depended on the humidity of the environment (Figures S11 and S13). The coefficient of friction was increased to 0.15 at RH ~ 50% but did not exceed 0.08 at RH ~ 9%. The wear of the MoS_2_ coating, however, was extremely weak in both humidity conditions (Figures S12 and S14). The coefficient of friction of the MoS_3_ coating was less dependent on moisture than was that of the MoS_2_ coating. After 400 cycles, the coefficient of friction was 0.1 and 0.14 at RH ~ 9% and RH ~ 50%, respectively. Apparently, an increase in the temperature to 22 °C caused some growth in the coefficient of friction in the MoS_3_ coating. A study of the optical images of the wear tracks after tribo-testing at RH ~ 9% and RH ~ 50% did not reveal a significant influence of humidity on the wear of this coating (Figures S12 and S14).

Thus, the differences in the local atomic packing of the MoS_2_ and MoS_3_ coatings caused a noticeable variation in their tribological properties both at low temperatures and at room temperature. The MoS_3_ coating was associated with the lowest coefficient of friction under adverse conditions, i.e., in the presence of an oxidizing environment at low temperatures and at room temperature. The wear of the MoS_2_ coating, however, was less pronounced; this was due to the good adhesion of the wear debris to the surface of the MoS_2_ coating. The adhesion of wear debris to the MoS_2_ coating on the track could have been due to the fact that the original amorphous MoS_2_ coating contained MoS_2_ clusters, which could easily reorient on the surface layer to form terrace-terminated MoS_2_ tribo-films.

The surfaces of these films interacted weakly with the surfaces of the steel counterbodies, so the exfoliated particles of the tribo-film did not adhere to the ball surface, but rather moved to another region of the track. The layered wear particles interacted with the oxidizing environment at the edges of layers, which caused the formation of metal oxide compounds. These compounds worsened the ductile properties of the MoS_2_-containing wear particles and had a negative impact on the coefficient of friction. These impacts increased the wear of the counterbody. As humidity decreased, the efficiency of the oxidation processes declined and the coefficient of friction of the MoS_2_ coating significantly diminished. The dependence of the tribological properties on air humidity is quite typical of MoS_2_-based coatings [51,52,53,54]. Even at low humidity (RH ~ 9%), a decrease in temperature to −100 °C promoted the adsorption of H_2_O molecules into the coating surface. Thus, the tribological properties of the coating were similar to the properties observed at 22 °C and RH ~ 50%.

Forming an MoS_2_ tribo-film on the surface of an amorphous MoS_3_ coating requires the removal of excess sulfur and atom transfer, which are the processes that contribute to the formation of a layered-type MoS_2_ structure. Lince et al. [34] assumed that, in the absence of oxygen and water, this is the result of thermally activated pyrolysis of MoS_3_. The necessary energy input is supplied by high local temperatures in the contact region due to mechanical energy that causes deformation in the MoS_3_ clusters. Reactions leading to the formation of Mo–O–S compounds are more likely in the presence of oxygen. According to the MRS study, these compounds were present in the wear debris of the MoS_3_ coating. There were, however, no obvious signs of an oxidation reaction in the coating material on the track. It can be assumed that, in the presence of water, a reaction occurred that is typical of the initial stage of the electrochemical process of hydrogen evolution on amorphous MoS*_x_*_≥2_ films. In this reaction, excess sulfur is removed because of the formation of H_2_S molecules [27,28]. The introduction of hydrogen into the MoS_3_ structure can lead to the reorganization of chemical bonds and the formation of MoS_2_-like clusters [40]. During the formation of MoS_2_ compounds in the area of contact between the MoS_3_ coating and the steel counterbody, this compound probably has a very defective structure, and thus it can interact effectively with the surface of the counterbody [51] and adhere to it. Therefore, in the area of contact between MoS_3_ and the steel counterbody, competing processes can take place, i.e., structural rearrangement (MoS_3_ to MoS_2_) and the adherence of Mo and S atoms to the counterbody. Adhesive interactions between the MoS_3_ surface layer and the steel ball can cause wear on this coating.

To activate the H_2_S molecule evolution reaction, it is necessary to electrically polarize local regions of the track surface, and thus produce negative potential. MoS*_x_* films have semiconductor properties [53]. In the contact area between the metal counterbody and the semiconductor, a rapid flow of electrons from the metal to the semiconductor is possible. This flow causes the formation of a built-in negative charge in the local regions of the MoS_3_ coatings. This charge repels (OH)^−^ molecules formed by the dissociative adsorption of H_2_O molecules. As a result, the flow of O-containing polarized molecules toward the MoS_3_ coating surface in the counterbody contact area may decrease. 

A comparison between the tribo-testing results of the MoS_3_ coatings and the MoS_4_ coatings showed that the tribo-chemical processes and the structural modification of MoS*_x_* materials in the steel counterbody contact area significantly depended on the concentration of sulfur. This concentration largely determined local atomic packing into nanoclusters. Understanding the mechanisms of wear of these materials also requires knowledge of their mechanical properties, which are currently being studied and will be discussed in future papers.

## 5. Conclusions

Reactive pulsed laser deposition of MoS*_x_* thin-film coatings from an Mo target in a standard configuration, which led to normal laser plume incidence on the substrate surface, made it possible to obtain sufficiently dense MoS*_x_* coatings if the pressure of the reactive H_2_S gas did not exceed 29 Pa. The selection of appropriate H_2_S pressures made it possible to obtain MoS_2_, MoS_3_, and MoS_4_ coatings. These coatings had an amorphous structure. Local atomic packing into nanoclusters, however, was different in each case. This difference became evident during the tribo-testing of the obtained coatings in an oxidizing environment (RH ~ 9%) at −100 °C.

The amorphous structure of MoS_2_ coatings contained clusters with a layered-type atomic packing characteristic of the 2H-MoS_2_ phase, as well as Mo–S_3_ linear clusters. This structure facilitated the formation of a terrace-terminated MoS_2_ tribo-film, which adhered weakly to the surface of the steel counterbody and held firmly to the track surface. As a result, the wear on this surface was minimal compared to that of MoS_3_ and MoS_4_. The adherence of wear debris to the track of the MoS_2_ coating was accompanied by oxidation, which caused an increased coefficient of friction (~0.16) and the most pronounced wear of the steel counterbody.

The amorphous structure of the MoS_3_ coating consisted of Mo_3_–S and Mo–S_3_ clusters connected as a polymer-like net. Atomic packing in the Mo_3_–S clusters was weakly ordered. The amorphous structure of the MoS_3_ coating went through a weak modification during counterbody sliding. This structure, however, could transform into a terrace-terminated MoS_2_ tribo-film. The transformation probably involved water molecules that could split apart in the field of charges in the MoS*_x_* semiconductor material and in the local areas of the counterbody. The effect of the water, which was, to some degree, beneficial, translated into the MoS_3_ coating having the lowest coefficient of friction (0.08). Tribo-induced changes in the MoS_3_ coating were accompanied by a transfer of the coating material to the counterbody; this led to layer-by-layer removal of the surface layers of the MoS_3_ coating and the relatively slow wear of the latter.

The MoS_4_ coating had an amorphous structure consisting of Mo_3_S_13_/Mo_3_S_12_ clusters with sufficiently perfect atomic packing. The sliding of the steel counterbody over that surface caused local packing disorder as well as the formation of an MoS_2_-containing tribo-film. The tribo-modification of the MoS_4_ coating structure caused the formation of edge-terminated MoS_2_ clusters, and the coefficient of friction of the MoS_4_ coating reached a value of 0.29. Thus, this coating was subject to the most intensive wear.

## Supplementary Materials

The following are available online at https://www.mdpi.com/2079-4991/10/4/653/s1, Figure S1: Possible local structures/atomic packings in amorphous MoS_x_ coatings with x~2, 3, and 4 (Mo atoms—blue, S atoms—yellow). Different types of S ligands that may present in polymerized amorphous MoS_x_ structures are indicated for Mo_3_S_12_ and Mo_3_S_13_ cluster unites in colored squares: green, terminal S2^2−^; red, apical S^2−^; blue, bridging S2^2−^; yellow, unsaturated S^2−^, Figure S2: Anton Paar TRB3 tribometer modified by the authors for friction testing of MoS_x_ thin-film coatings at low temperatures, Figure S3: Chemical mapping of S, Mo, Fe, and Cr over the surface of different samples obtained by RPLD of MoS_x_ coatings on the polished steel substrates, Figure S4: RBS spectra for thin MoS_x_ films obtained by RPLD on Si substrates at different pressures of H_2_S gas, Figure S5: Time-of-flight (TOF) signals of ion pulses detected by an ion probe during the pulsed laser ablation of a Mo target at different pressures of H_2_S gas, Figure S6: High resolution TEM and SAED patterns for thin MoS_4_ film obtained by RPLD. The time of in situ electron beam irradiation of the film in the microscope was (a) 2 and (b) 10 min. The e-beam irradiation caused the local modification/crystallization of the film. Lattice spacing in the e-beam induced nanophase was ~0.26 nm. The nature of this nanophase has not been established. It can be assumed that the electron irradiation caused the desorption of sulfur atoms and, as a result, the local formation of the Mo_2_S_3_ nanocrystals in the amorphous MoS_x_ matrix. The high resolution TEM image of this compound clearly showed the lattice spacing of 0.255 nm [1], Figure S7: 2D images and profiles of the wear scar edge for the different MoS_x_ coatings measured after tribo-testing at −100 °C in an oxidizing environment. Red lines show the cross sections of wear scars at indicated places; blue lines show the depths of the tracks alone the direction of ball sliding. For the MoS_2_ coating, the profilometry studies revealed the uneven wear along the entire track, therefore, sufficiently accurate profiling along the track could not be done, Figure S8: Distribution of elements (Mo, S, Fe, Cr, and O) across the central part of the wear track on the MoS_2_ coating after tribo-testing at −100 °C in an oxidizing environment, Figure S9: Distribution of elements (Mo, S, Fe, Cr, and O) across the central part of the wear track on the MoS_3_ coating after tribo-testing at −100 °C in an oxidizing environment, Figure S10: Distribution of elements (Mo, S, Fe, Cr, and O) across the central part of the wear track on the MoS_4_ coating after tribo-testing at −100 °C in an oxidizing environment, Figure S11: Friction curves of tests conducted at 22 °C for different RPLD MoS_x_ coatings in an argon‒air mixture (RH~9%), Figure S12: Optical images of the wear tracks for different MoS_x_ coatings after tribo-testing at 22 °C in an argon‒air mixture (RH~9%), Figure S13: Friction curves of tests conducted at 22 °C for different RPLD MoS_x_ coatings in air (RH~50%), Figure S14: Optical images of the wear tracks for different MoS_x_ coatings after tribo-testing at 22 °C in air (RH~50%).
