# Supplementary material for: Reactive Pulsed Laser Deposition of Clustered-Type MoSx (x ~ 2, 3, and 4) Films and Their Solid Lubricant Properties at Low Temperature
Source: Nanomaterials (Basel). 2020 Apr 1;10(4):653. doi: 10.3390/nano10040653 (PMC7221609; doi:10.3390/nano10040653)
Supplement: Supplementary file 1 [file nanomaterials-10-00653-s001.pdf]

Supplementary Materials

Reactive pulsed laser deposition of clustered-type MoS_x (x~2, 3, and 4) films and their solid lubricant properties at low temperature

V. Fominski [1,*], M. Demin[2], V. Nevolin[1], D. Fominski[1], R. Romanov [1], M. Gritskevich[1], N. Smirnov[3]

[1]National Research Nuclear University MEPhI (Moscow Engineering Physics Institute), Moscow, Kashirskoe sh., 31, Russia, 115409

[2]Russia Immanuel Kant Baltic Federal University, Kaliningrad, A. Nevskogo str. 14, Russia, 236016

[3]Mechanical Engineering Research Institute of the Russian Academy of Sciences, Moscow, Bardina Ulitsa 4, Russia, 119334

Local atomic structures/cluster unites of amorphous MoS_x materials with varied S content

$MoS_{x\sim2}$

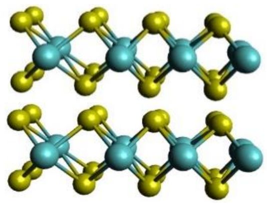

Lamellar/layered-type structure

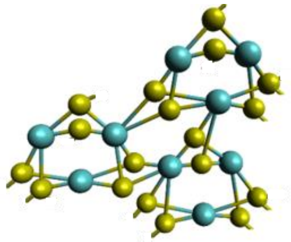

Mo_3-S (Mo_3S_6) cluster units

$MoS_{x\sim3}$

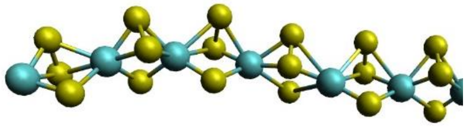

Linear chain of Mo-S_3 (MoS_3) cluster nits

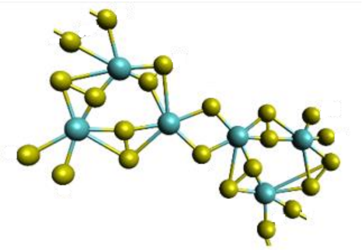

Mo_3-S (Mo_3S_9) cluster units

$MoS_{x\sim4}$

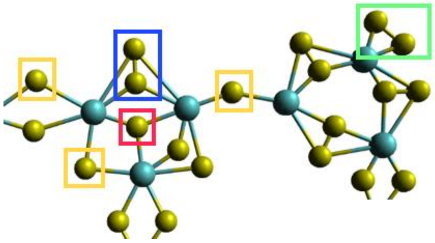

Polymer-like chain consisted of Mo_3S_{12} and Mo_3S_{13} cluster units

Figure S1. Possible local structures/atomic packings in amorphous MoS_x coatings with x~2, 3, and 4 (Mo atoms – blue, S atoms – yellow). Different types of S ligands that may present in polymerized amorphous MoS_x structures are indicated for Mo_3S_{12} and Mo_3S_{13} cluster unites in colored squares: green, terminal S_2^{2-}; red, apical S^{2-}; blue, bridging S_2^{2-}; yellow, unsaturated S^{2-}.

Tribometer for friction testing of thin-film coatings by pin-on-dis method at various conditions

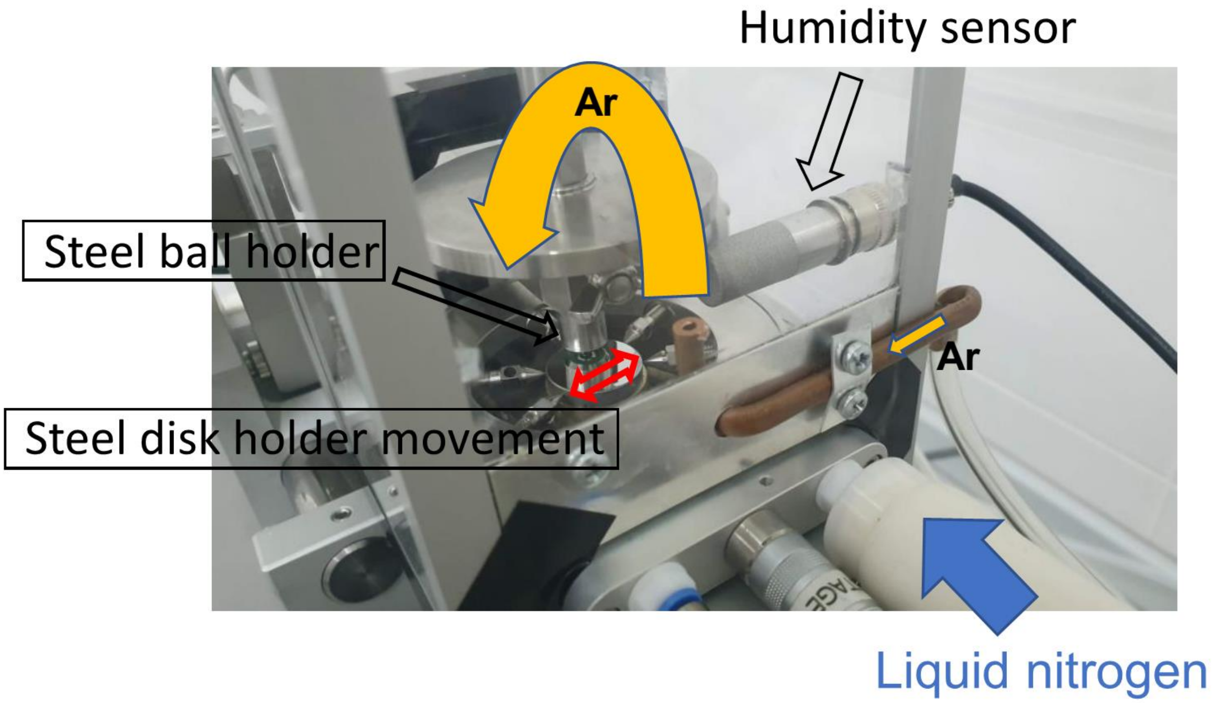

Figure S2. Anton Paar TRB3 tribometer modified by the authors for friction testing of MoS_x thin-film coatings at low temperatures.

Composition and surface distribution of elements for MoS_x thin-film coatings obtained by RPLD

MoS_2

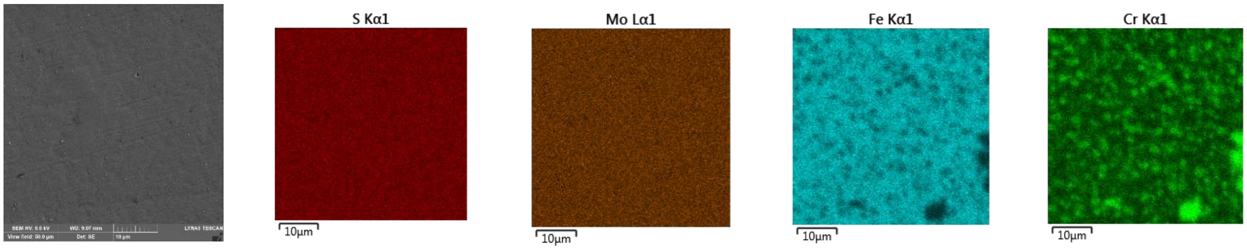

MoS_3

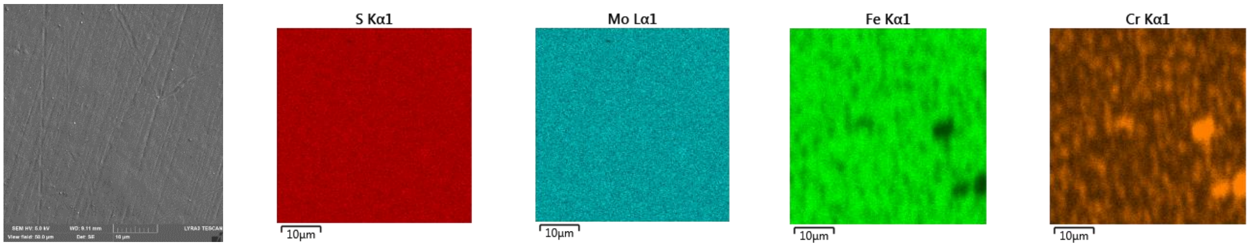

MoS_4

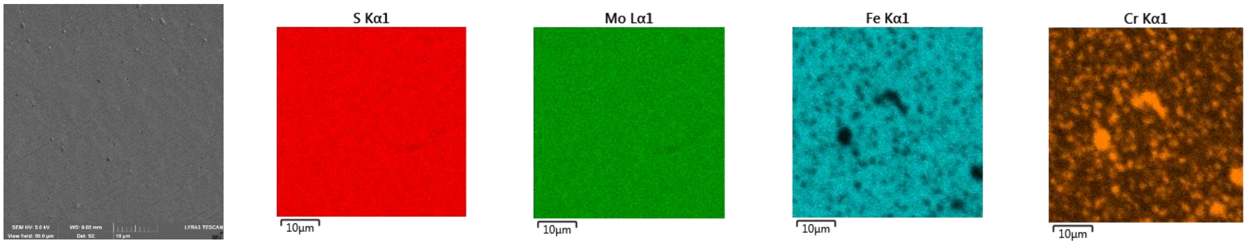

Figure S3. Chemical mapping of S, Mo, Fe, and Cr over the surface of different samples obtained by RPLD of MoS_x coatings on the polished steel substrates.

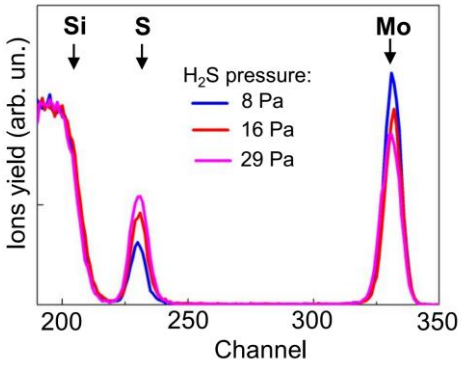

Figure S4. RBS spectra for thin MoS_x films obtained by RPLD on Si substrates at different pressures of H_2S gas.

Monitoring of laser-initiated ion fluxes bombarded the MoS_x coating during RPLD at different pressures of H_2S gas

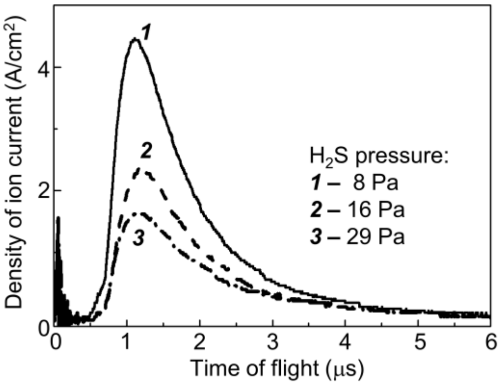

Figure S5. Time-of-flight (TOF) signals of ion pulses detected by an ion probe during the pulsed laser ablation of a Mo target at different pressures of H_2S gas.

Structural study of MoS_4 film obtained by RPLD

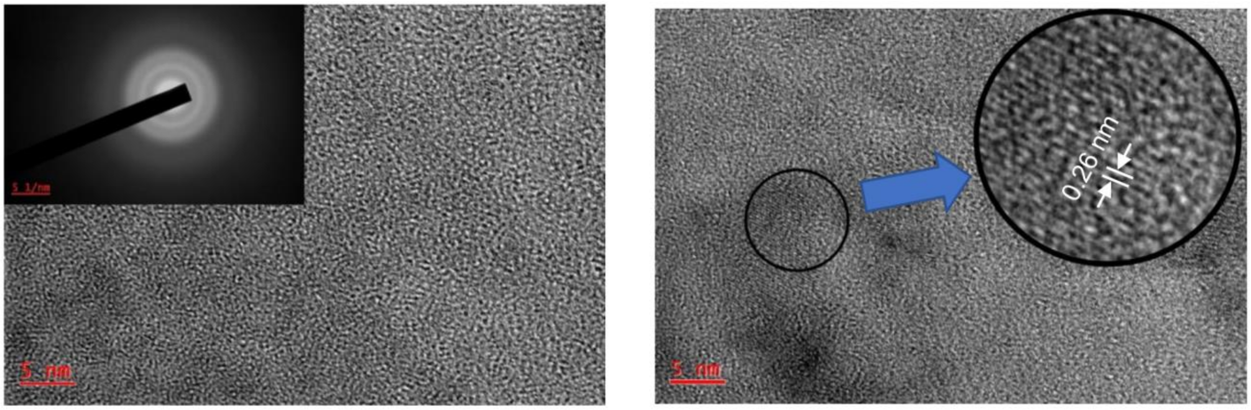

(**a**) (**b**)

Figure S6. High resolution TEM and SAED patterns for thin MoS_4 film obtained by RPLD. The time of *in situ* electron beam irradiation of the film in the microscope was (**a)** 2 and (**b**) 10 minutes. The e-beam irradiation caused the local modification/crystallization of the film. Lattice spacing in the e-beam induced nanophase was ~0.26 nm. The nature of this nanophase has not been established. It can be assumed that the electron irradiation caused the desorption of sulfur atoms and, as a result, the local formation of the Mo_2S_3 nanocrystals in the amorphous MoS_x matrix. The high resolution TEM image of this compound clearly showed the lattice spacing of 0.255 nm [1].

Distribution of wear along the tracks for MoS_2, MoS_3, and MoS_4 coatings

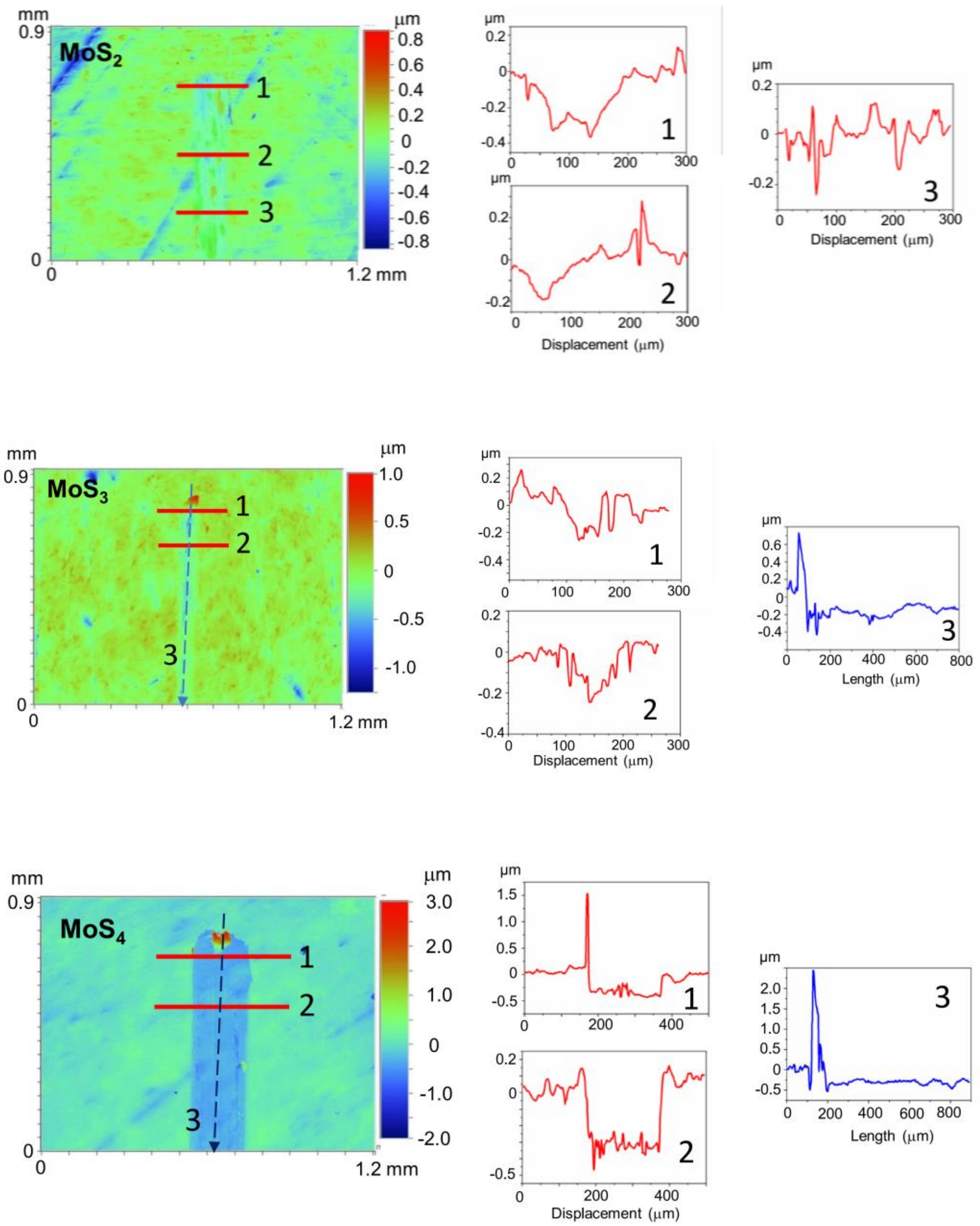

Figure S7. 2D images and profiles of the wear scar edge for the different MoS_x coatings measured after tribo-testing at -100°C in an oxidizing environment. Red lines show the cross sections of wear scars at indicated places; blue lines show the depths of the tracks alone the direction of ball sliding. For the MoS_2 coating, the profilometry studies revealed the uneven wear along the entire track, therefore, sufficiently accurate profiling along the track could not be done.

Wear of RPLD MoS_x coatings caused by tribotesting at -100°C

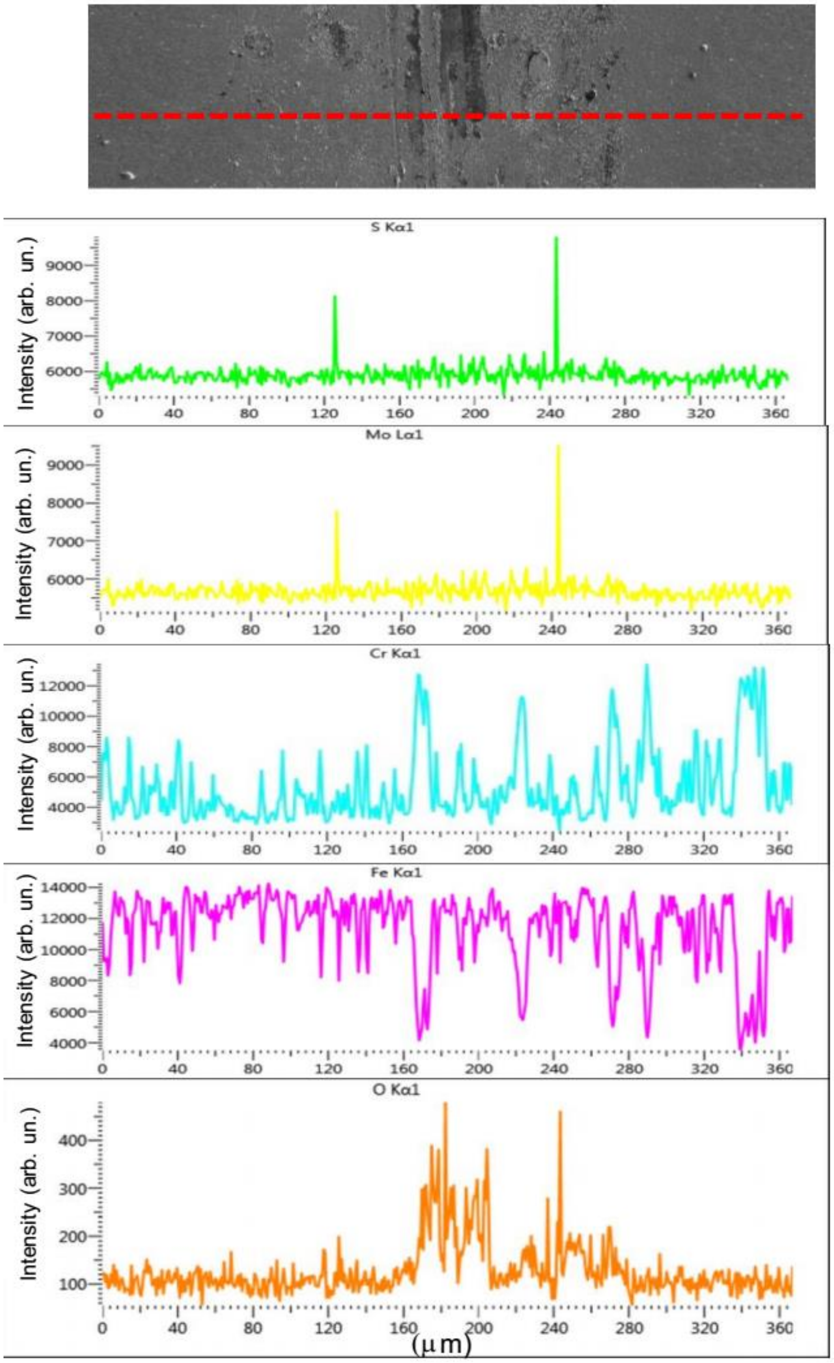

Figure S8. Distribution of elements (Mo, S, Fe, Cr, and O) across the central part of the wear track on the MoS_2 coating after tribo-testing at -100°C in an oxidizing environment.

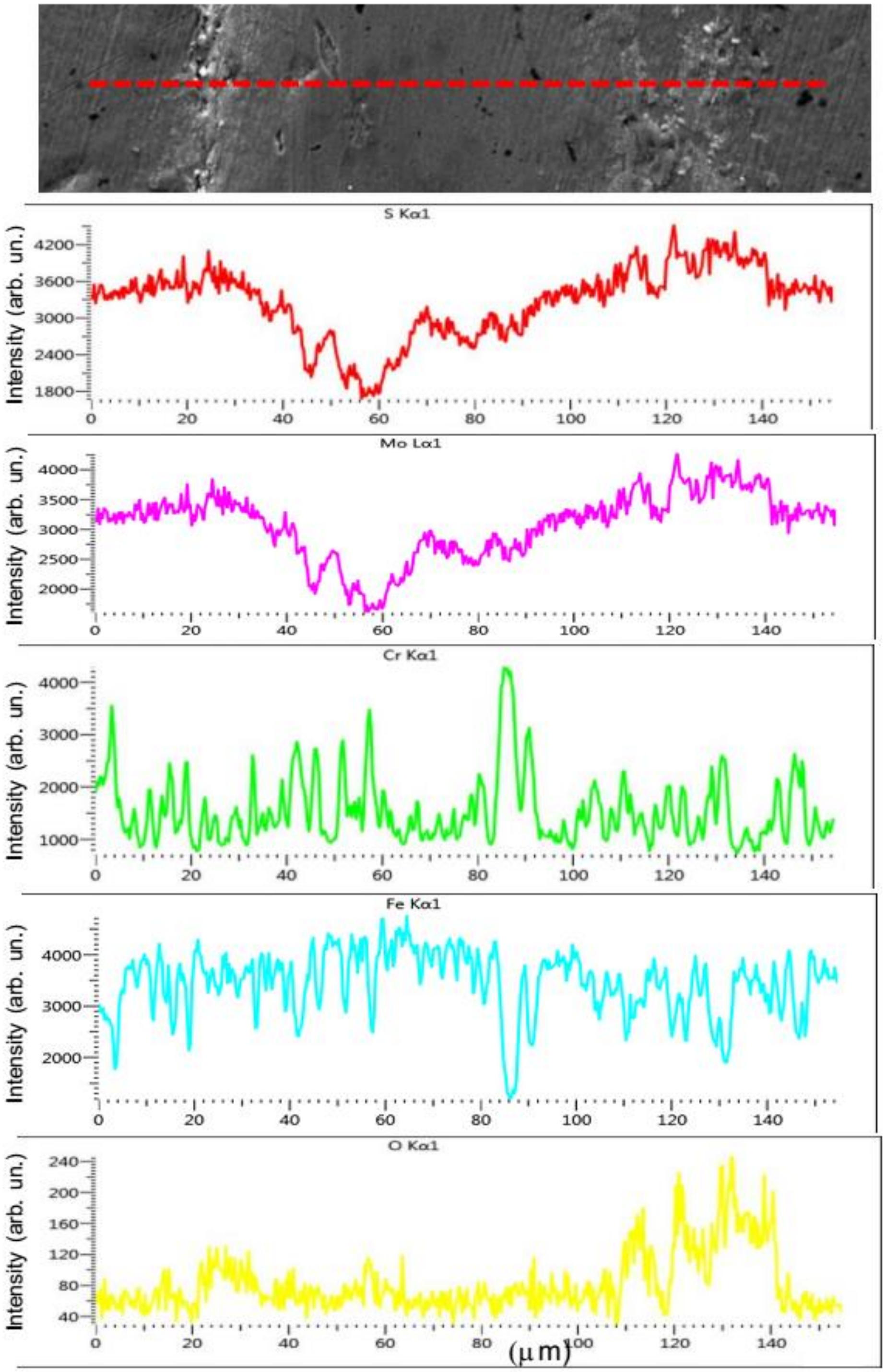

Figure S9. Distribution of elements (Mo, S, Fe, Cr, and O) across the central part of the wear track on the MoS_3 coating after tribo-testing at -100°C in an oxidizing environment.

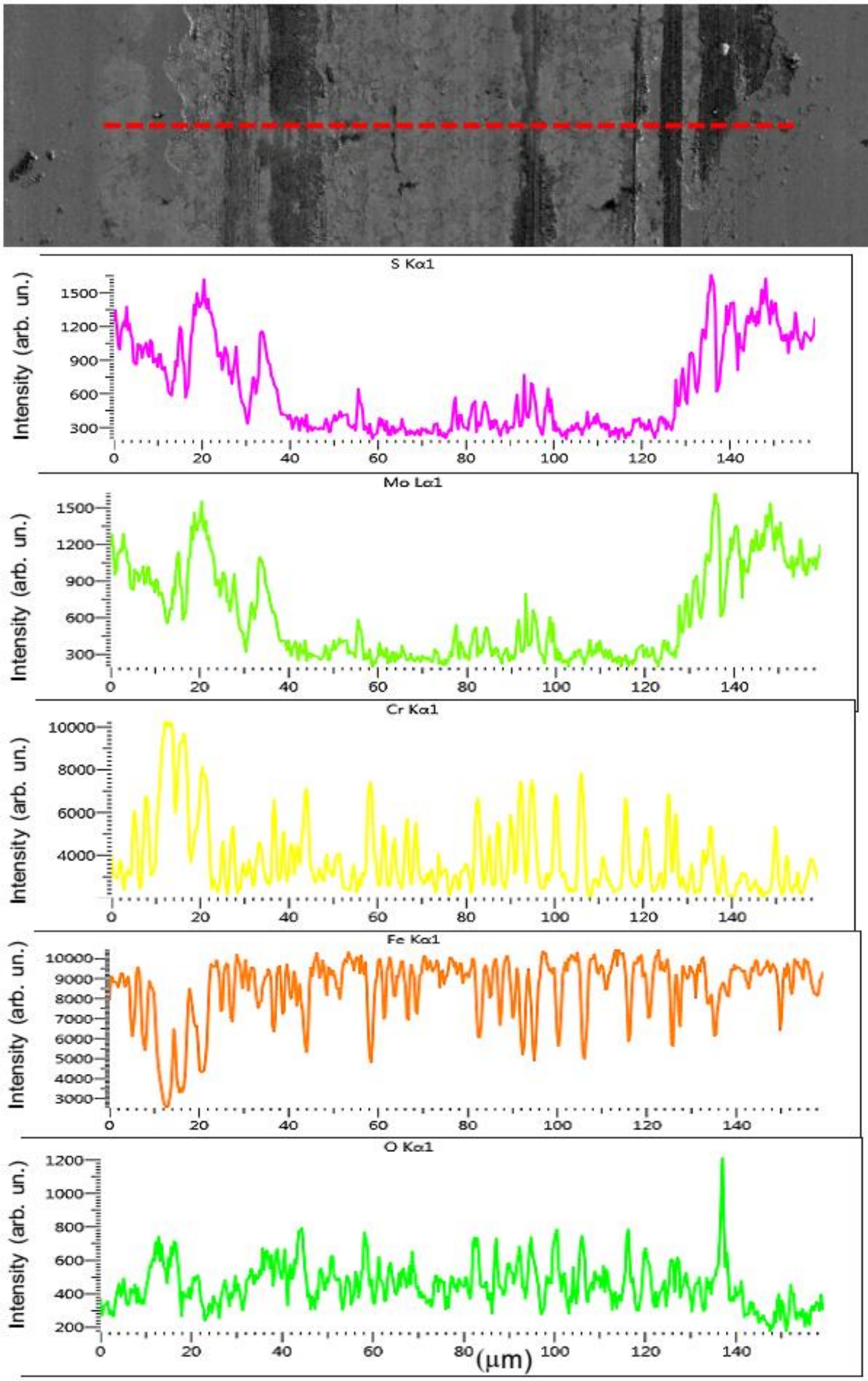

Figure S10. Distribution of elements (Mo, S, Fe, Cr, and O) across the central part of the wear track on the MoS_4 coating after tribo-testing at -100°C in an oxidizing environment.

Tribological properties of RPLD MoS_x coatings at 22°C

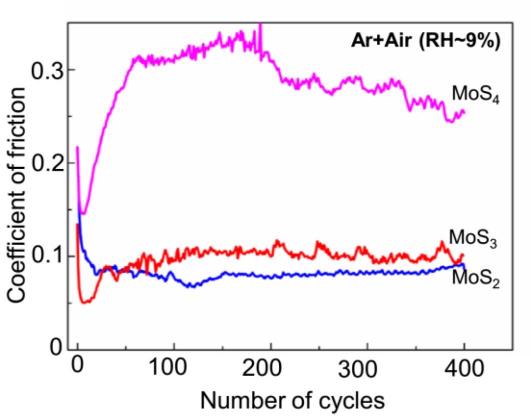

Figure S11. Friction curves of tests conducted at 22°C for different RPLD MoS_x coatings in an argon–air mixture (RH~9%).

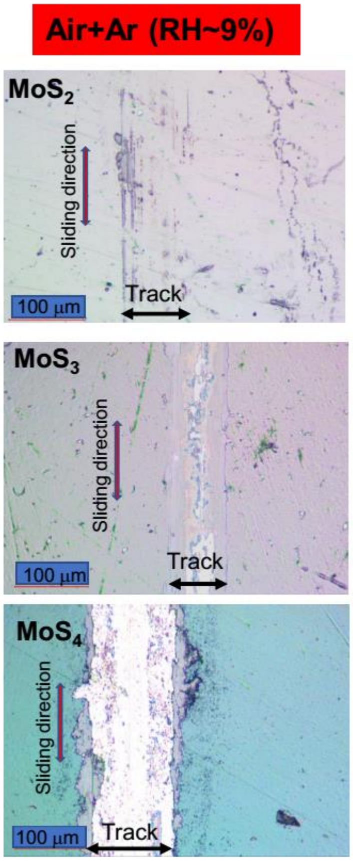

Figure S12. Optical images of the wear tracks for different MoS_x coatings after tribo-testing at 22°C in an argon–air mixture (RH~9%).

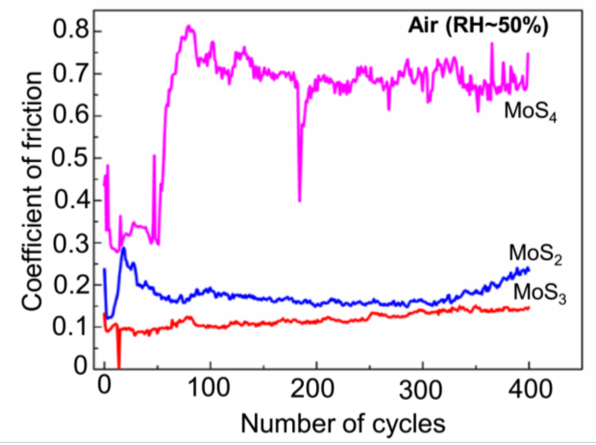

Figure S13. Friction curves of tests conducted at 22°C for different RPLD MoS_x coatings in air (RH~50%).

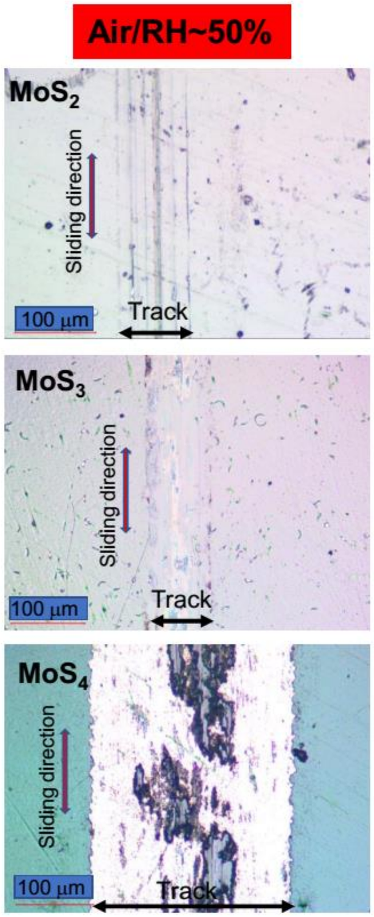

Figure S14. Optical images of the wear tracks for different MoS_x coatings after tribo-testing at 22°C in air (RH~50%).

Reference

1. X. Zhou, W. Zhao, J. Pan, Y. Fang, F. Wang, F. Huang, Urchin-like Mo_2S_3 prepared via a molten salt assisted method for efficient hydrogen evolution, Chem. Commun. 2018, 54, 12714.
